# The synaptonemal complex central element SCEP3 interlinks synapsis initiation and crossover formation in *Arabidopsis thaliana*

**DOI:** 10.1038/s41477-025-02030-9

**Published:** 2025-06-27

**Authors:** Chao Feng, Jana Lorenz, Steven Dreissig, Veit Schubert, Baicui Wang, Franziska Hartmann, Maria Cuacos, Nadia Fernández-Jiménez, Ziliang Zhao, Christian Eggeling, Amanda Souza Câmara, Axel Himmelbach, Stefan Heckmann

**Affiliations:** 1 Leibniz Institute of Plant Genetics and Crop Plant Research, Seeland, Germany; 2 Institute of Agricultural and Nutritional Sciences, Martin-Luther-University Halle-Wittenberg, Halle (Saale), Germany; 3 Departamento de Genética, Fisiología y Microbiología, Facultad de Ciencias Biológicas, Universidad Complutense de Madrid, Madrid, Spain; 4 Leibniz Institute of Photonic Technologies, Jena, Germany; 5 Institute of Applied Optics and Biophysics, Friedrich-Schiller-Universität Jena, Jena, Germany; 6 Leibniz Centre for Photonics in Infection Research, Jena, Germany

**Keywords:** Plant cell biology, Plant reproduction, Plant genetics, Plant development

## Abstract

The synaptonemal complex (SC) forms between homologous chromosomes during meiosis. In *Arabidopsis thaliana*, its central region (CR) is composed of the transverse filament protein ZYP1 and the central element proteins SCEP1 and SCEP2. Here we identify SCEP3 as a CR protein that is evolutionarily conserved across plant species. SCEP3 spatiotemporally overlaps with other CR proteins and localizes to the SC CR. The loss of SCEP3 prevents SC assembly, abolishes crossover (CO) assurance and interference, and eliminates sex-specific differences in CO rates (heterochiasmy) through increased CO in females. SCEP3 is required for a subset of COs in SC-deficient mutants, such as *zyp1*. Although SCEP3 physically interacts with ZYP1, it loads independently of other CR proteins. We propose that SCEP3 may associate with certain recombination intermediates, stabilizing them and/or recruiting additional factors, such as ZYP1, to a subset of these intermediates, thereby promoting and interlinking SC assembly and CO formation.

## Abstract (web summary)

This study identifies SCEP3 as a conserved central element of the plant synaptonemal complex. SCEP3 is critical for synapsis, heterochiasmy and crossover interference. SCEP3 also promotes a subset of crossover events.

## Main

Meiotic homologous recombination assures genetic diversity in gametes^1^. The repair of programmed DNA double-strand breaks (DSBs) into interhomologue crossovers (COs) involves numerous proteins and several consecutive steps^2,3^. In most species, COs are divided into two classes. Class I COs, promoted by ZMM proteins (Zip1-4, Mer3 and Msh4/5)^4,5^ and MutL-γ (Mlh1/3)^6^, are interference-sensitive (one CO limits the probability of other COs nearby)^7^. Class II COs are insensitive to interference and form a minority in most species, including *Arabidopsis thaliana*, where their formation depends in part on MUS81 (refs. ^8,9^).

Meiotic recombination occurs during prophase I. Sister chromatids are initially organized into a linear loop–base array by a proteinaceous structure called the meiotic chromosome axis^1^. In *Arabidopsis*, the axis consists of ASY1 (refs. ^10,11^), ASY3 (ref. ^12^), ASY4 (ref. ^13^) and cohesion-associated proteins including REC8 (ref. ^14^). Initial repair of a large number of DSBs (~200 in *Arabidopsis*) leads to numerous meiotic recombination intermediates, including early interhomologue associations^15^. Upon installation of the transverse filament protein ZYP1, aligned chromosomes become physically connected at ~200 nm (ref. ^16^), leading to the formation of the tripartite synaptonemal complex (SC)^1^. In *Arabidopsis*, homologue alignment/pairing, albeit at a larger distance at ~300–400 nm, is found even in the absence of the SC^16–18^.

The SC structure, two lateral elements flanking a central region (CR), is conserved across species and regulates the number and distribution of COs^1^. Across species, the CR is composed of transverse filament proteins (such as Zip1 in *Saccharomyces cerevisiae*^19^, C(3)G in *Drosophila melanogaster*^20^, SYP-1/5/6 in *Caenorhabditis elegans*^21–23^, SYCP1 in *Mus musculus*^24^ and ZYP1 (ZYP1a/b) in *A. thaliana*^16,17,25^) and central element (CE) proteins (such as Ecm11 and Gmc2 in budding yeast^26^; Corona and Corolla in *Drosophila*^27,28^; SYP-2/3/4 and SKR-1/2 in worms^29–32^; SYCE1/2/3, TEX12 and SIX6OS1 in mice^33–37^; and SCEP1/2 in *Arabidopsis*^18^).

In most species, including flies, mice, worms and *Sordaria*, the absence of the SC impairs CO formation^15^. In plants, the SC is dispensable for CO formation in *Arabidopsis* and rice^16–18,38^ but probably required in barley^39^. Studies of *Arabidopsis* CR mutants suggest that the SC is critical for CO assurance, heterochiasmy and CO interference^16–18,40^. On the basis of *Arabidopsis* data, the coarsening of the ZMM protein HEI10 in the frame of the SC was proposed as the basis for CO interference^40,41^. However, the SC is not required for implementing CO interference in budding yeast or *Sordaria*^1^.

Interactions among CR proteins are linked to SC assembly—for example, in budding yeast, Ecm11–Gmc2 promotes Zip1 polymerization^26^, and in mice, SYCP1 tetramers remodelled by SYCE3 form a SYCP1–SYCE3 complex, and SYCE3 also interacts with TEX12–SYCE2 and SYCE1–SIX6OS1 (ref. ^42^). In budding yeast, SC assembly and CO formation are coupled via the interaction of Ecm11–Zip4 (in mouse via orthologues TEX12–TEX11)^43^. In worms, CO formation is regulated by SYP-4’s carboxy terminus, possibly through the recruitment of ZHP-3 (an orthologue of *Arabidopsis* HEI10) to the SC^44^. In *Arabidopsis*, SCEP1 and SCEP2 directly interact, while neither interacts with ZYP1 or ZIP4 (ref. ^18^), and HEI10 coarsening in the SC may underlie CO interference^40,41^. It is unclear whether further CE proteins exist in *Arabidopsis* that form a complex with ZYP1 and/or interconnect SC assembly and CO formation.

We previously identified proteins in proximity to ASY1 and ASY3 via proximity proteomics^45^. Here we functionally characterize the candidate ATC21, renamed SCEP3. SCEP3 is a CE protein that is conserved in plants and is probably the structural orthologue of SYP-4 in worms and SIX6OS1 in mammals^31,36^, suggesting conservation across kingdoms.

## Results

### SCEP3 is required for synapsis and obligate CO formation

**Fig. 1:**
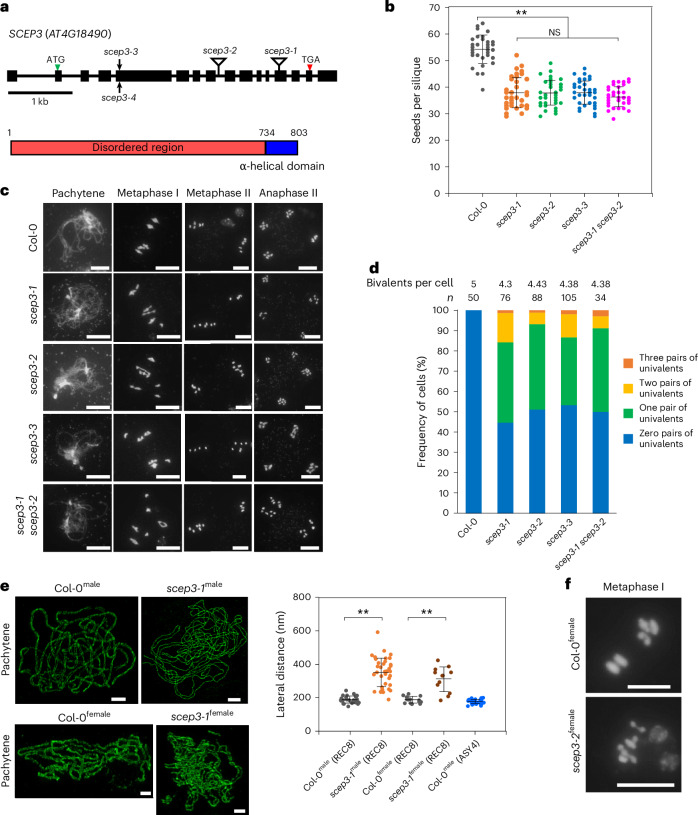
Identification of *SCEP3* and phenotypic analysis of *scep3* mutants. **a**, Gene model of *SCEP3* (AT4G18490; confirmed by Sanger sequencing of flower bud complementary DNA), including exons (black boxes) and introns (black lines), and a schematic depiction of the SCEP3 protein. The locations of mutant alleles are indicated: *scep3-1* (initially named *atc21-1* (ref. ^44^)) and *scep3-2* (transfer DNA insertions within exon 13 and intron 8, respectively) as well as *scep3-3* and *scep3-4* (CRISPR–Cas9-based mutagenesis); *scep3-4* is in Ler-0, while all other alleles are in the Col-0 background. **b**, Seeds per silique in the WT (54.33 ± 5.38, *n* = 40), *scep3-1* (37.98 ± 5.76, *n* = 40), *scep3-2* (37.9 ± 4.75, *n* = 40), *scep3-3* (38.05 ± 4.44, *n* = 40) and *scep3-1* *scep3-2* (36.33 ± 3.59, *n* = 40). No significant differences were found among the *scep3* mutants (*P* = 0.30). However, all *scep3* mutants produced significantly fewer seeds than the WT (*P* < 1 × 10^−7^). **c**, Male meiotic chromosome behaviour (scale bars, 10 μm; DNA counterstained with DAPI is shown in grey) in the WT and *scep3* mutants. **d**, Frequency of cells with zero to three pairs of univalents, including the average bivalent number per cell (*n* is the number of cells analysed) in the WT and *scep3* mutants. **e**, Left, 3D-SIM analysis of REC8 immunolocalization in male and female meiocytes of Col-0 and *scep3-1*. Along synapsed chromosomes, the two parallel lateral elements exhibit an average distance of 188 ± 21.6 nm (range, 148–243 nm; *n* = 26) in WT males and 187 ± 19.4 nm (range, 162–223 nm; *n* = 15) in WT females. In *scep3-1*, within regions of alignment, the average distance increased to 352 ± 87.5 nm (range, 189–592 nm; *n* = 37) in males and 311 ± 74.6 nm (range, 184–423 nm; *n* = 12) in females, showing greater variation. Single-slice images were used to measure the distances between two aligned axes. Scale bars, 2 μm. Right, quantification of aligned axis distances (the measurement points were randomly selected) in both Col-0 and *scep3-1*. ASY4-labelled lateral elements exhibit an average distance of 176 ± 13.4 nm (range, 149–197 nm; *n* = 28) in WT males. **f**, Female meiotic chromosome spreads of Col-0 and *scep3-2* (DAPI-stained DNA is shown in grey). The experiments were repeated three times with similar results. Scale bars, 10 μm. Distinct plants from each mutant line (or the WT) were used for seed counting and chromosome spread analysis. Significance was evaluated using one-way ANOVA with post hoc Bonferroni multiple comparison. The data are presented as mean ± s.d. **P < 0.01. NS, not significant. Source data

**Extended Data Fig. 1:**
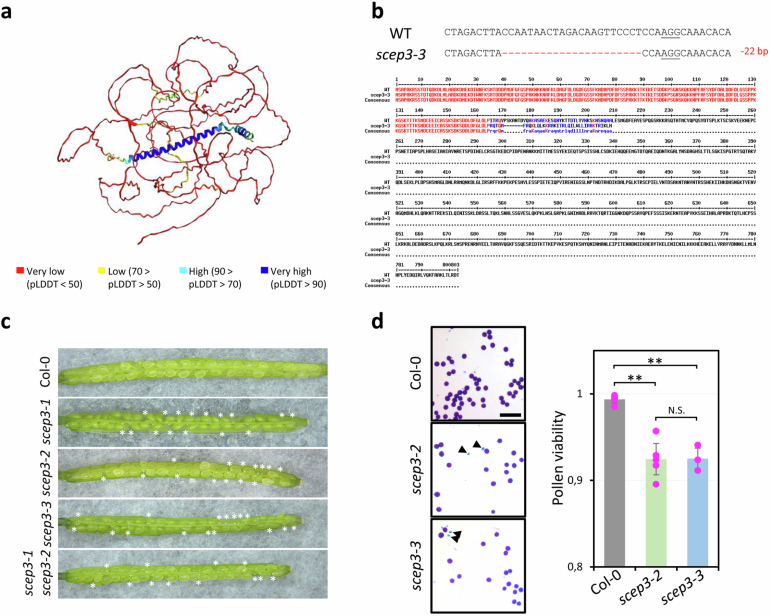
Isolation of scep3-3 and phenotypic analysis of scep3 alleles. **a**, SCEP3 full-length protein structure prediction by AlphaFold2^46^. **b**, Isolation of *scep3-3*. Top: CRISPR/Cas9-mediated 22 bp deletion in exon 5 resulting in a predicted truncated protein of 201 AA; PAM sequence underlined and deleted bases as red dashes. Below: Alignment of SCEP3 WT and SCEP3-3 proteins. **c**, Seed abortion (indicated by asterisks) in the WT and *scep3* alleles. **d**, Alexander staining of pollen grains (arrowheads indicate non-viable ones in blue; scale bar, 100 µm) and quantification of pollen viability in Col-0 (0.99 ± 0.01, n = 5), *scep3-2* (0.92 ± 0.02, n = 5) and *scep3-3* (0.93 ± 0.01, n = 3). Significance evaluated by one-way ANOVA with post-hoc Bonferroni multiple comparison (Col-0 and *scep3-2*, *P* = 1.26×10^−4^; Col-0 and *scep3-3*, *P* = 4.48×10^−4^; *scep3-2* and *scep3-3*, *P* = 1). Data are presented as mean ± standard deviation (s.d.). **P < 0.01. N.S., not significant. Source data

*SCEP3* (AT4G18490) has 16 exons, encodes a protein of 803 amino acids (Fig. 1a) and is highly expressed in young flower buds^46^. According to structural prediction by AlphaFold2 (ref. ^47^), the last 70 amino acids at the C terminus form an α-helical domain, while the remaining 733 amino acids are disordered (Extended Data Fig. 1a). No obvious developmental differences were observed in any *scep3* mutant (*scep3-1*, *scep3-2*, *scep3-3* and *scep3-1* *scep3-2*; Fig. 1a and Extended Data Fig. 1b) compared to the wild type (WT), except for slightly shorter siliques with seed gaps (Extended Data Fig. 1c), reduced seed fertility (Fig. 1b) and decreased pollen viability (Extended Data Fig. 1d). In the WT, chromosomes are fully synapsed at pachytene, five bivalents are invariably observed at metaphase I and chromosome segregation is balanced (Fig. 1c,d). In *scep3*, pachytene chromosomes appeared as pairs of parallel threads, indicating the absence of synapsis despite chromosome alignment/pairing (Fig. 1c), and ~50% of cells displayed one to three pairs of univalents (failure to form the obligate CO), leading to unequal chromosome segregation (Fig. 1c,d). Moreover, minimum chiasmata numbers (rod- and ring-shaped bivalents are scored as one and two chiasmata, respectively) in *scep3* are reduced to ~70–75% of WT levels (Supplementary Table 1). Three-dimensional structural illumination microscopy (3D-SIM) inter-axis measurements confirmed that in *scep3* chromosomes align/pair, albeit at a greater and more variable distance than in the WT (Fig. 1e). As in males, during *scep3* female meiosis, achiasmatic and asynaptic yet aligned/paired chromosomes were found (Fig. 1e,f).

### SCEP3 is found at the CR of the SC

**Fig. 2:**
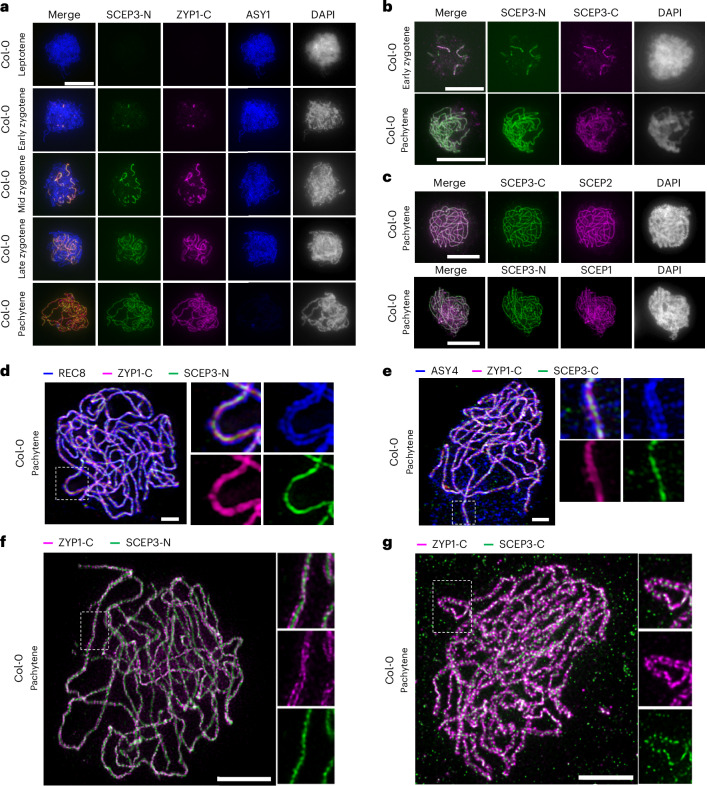
Localization of SCEP3 at the CR of the SC. **a**–**c**, Immunolocalization in the WT of SCEP3-N, ZYP1-C and ASY1 during prophase I (**a**); SCEP3-C and SCEP3-N during zygotene and pachytene (**b**); and SCEP3-C with SCEP2 or SCEP3-N with SCEP1 at pachytene (**c**). DAPI-stained DNA is shown in grey. **d**,**e**, 3D-SIM of a pachytene nucleus in the WT immunolabelled with REC8, ZYP1-C and SCEP3-N (**d**) or ASY4, ZYP1-C and SCEP3-C (**e**). **f**,**g**, STED microscopy of a WT pachytene nucleus immunolabelled with ZYP1-C and SCEP3-N (**f**) or ZYP1-C and SCEP3-C (**g**). Scale bars, 10 μm in **a**–**c**, 2 μm in **d**–**g**. All experiments were repeated at least two times with similar results. Source data

**Extended Data Fig. 2:**
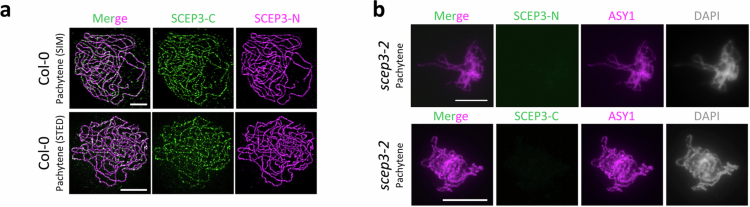
Both SCEP3-N and SCEP3-C antibodies localize to the SC and are specific for SCEP3 (signal absence in scep3). Immunolocalization in pachytene nuclei of **a**, SCEP3-N and SCEP3-C in Col-0 (Images acquired by 3D-SIM or STED; Scale bar, 2 μm), and of **b**, SCEP3-N or SCEP3-C together with ASY1 in *scep3-2*. DNA counterstained with DAPI in gray. The experiments were repeated three times with similar results. Scale bar, 10 μm.

To dissect the spatiotemporal localization of SCEP3, antibodies raised against its amino (SCEP3-N) or C terminus (SCEP3-C) were employed. Using SCEP3-N, SCEP3 is initially detected during early zygotene forming a limited number of foci (Fig. 2a). The foci numbers increase progressively, with initial SCEP3 stretches during zygotene and full polymerization at pachytene. During all stages, SCEP3 colocalizes with ZYP1 (Fig. 2a). SCEP3-C overlaps with SCEP3-N during zygotene/pachytene, albeit less continuously, leading to a patchier signal (Fig. 2b), as also revealed by both 3D-SIM and stimulated emission depletion (STED) microscopy of pachytene nuclei (Extended Data Fig. 2a). In any case, using SCEP3-C and SCEP3-N, SCEP3 also colocalizes with the CE proteins SCEP1 and SCEP2 at the SC (Fig. 2c). The absence of SCEP3-N and SCEP3-C signals in *scep3* confirms the specificity of both antibodies (Extended Data Fig. 2b).

Using 3D-SIM, the following SC organization was detected at pachytene: REC8 and ASY4 each form two parallel lines separated by approximately 188 and 176 nm, respectively (Figs. 1e and 2d,e). Between these lines, ZYP1-C (its C termini localize towards the chromosome axis^16^) appears as either two distinct lines or a single bright line. Both SCEP3-N and SCEP3-C are centrally located within the single or two ZYP1-C lines (Fig. 2d,e). A similar *Arabidopsis* SC organization—that is, REC8 axes at a distance of 175–213 nm, two lines of ZYP1-C in between and between those centrally located CE proteins SCEP1/2—was found using STED microscopy^16,18^. To further investigate whether SCEP3 is indeed a CE protein within the SC CR, STED microscopy was performed. Similar to the two known CEs SCEP1/2 (ref. ^18^), both SCEP3-N and SCEP3-C were located centrally between the two ZYP1-C lines (Fig. 2f,g). Together, SCEP3’s localization to the SC CR supports its role as a CE protein. Hereafter, unless otherwise specified, SCEP3-N was employed.

### SCEP3 is crucial for SC assembly and loads independently of the SC

**Fig. 3:**
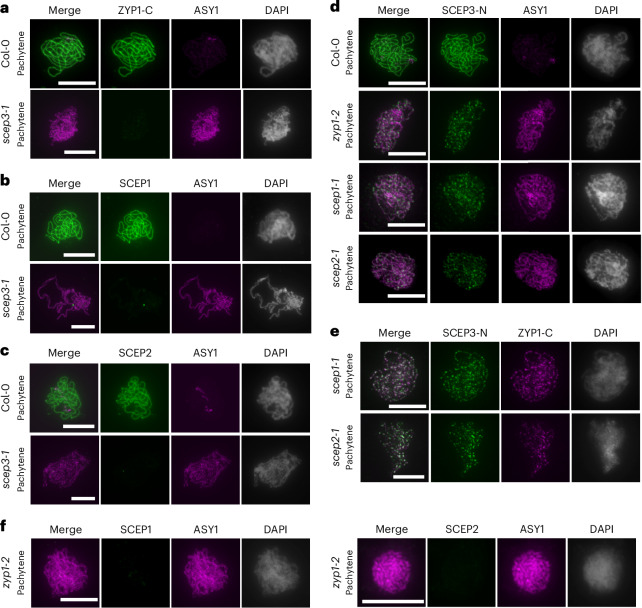
SCEP3 is critical for SC assembly, and its localization is independent of other CR proteins. **a**–**c**, Immunolocalization in WT and *scep3-1* pachytene nuclei of ZYP1-C and ASY1 (**a**), SCEP1 and ASY1 (**b**), and SCEP2 and ASY1 (**c**). **d**–**f**, Immunolocalization of SCEP3-N and ASY1 in the WT, *zyp1-2*, *scep1-1* and *scep2-1* (**d**); SCEP3-N and ZYP1-C in *scep1-1* and *scep2-1* (**e**); and SCEP1 or SCEP2 and ASY1 in *zyp1-2* (**f**). DAPI-stained DNA is shown in grey. All experiments were repeated three times with similar results. Scale bars, 10 µm.

In the WT, all CR proteins fully polymerize at pachytene, while ASY1 gets depleted from synapsed regions (Fig. 3a–d). In *scep3-1* pachytene(-like) nuclei, no chromosome-associated ZYP1, SCEP1 or SCEP2 foci are detected (Fig. 3a–c), indicating the absence of SC assembly in *scep3-1* and suggesting that chromosomal loading of ZYP1, SCEP1 and SCEP2 is SCEP3-dependent. The absence of synapsis is further reflected by the persistence of ASY1 at pachytene(-like) stages^16,18^ (Fig. 3a–c). In *zyp1-2*, *scep1-1* and *scep2-1* pachytene(-like) nuclei, axis-associated SCEP3 foci are found (Fig. 3d), indicating that SCEP3 loads independently of SC formation and of other CR proteins. Additionally, ZYP1 colocalizes with SCEP3 in *scep1-1* and *scep2-1* early pachytene(-like) nuclei (Fig. 3e), whereas neither SCEP1 nor SCEP2 is detected in *zyp1-2* (Fig. 3f). This suggests that ZYP1 localization depends on SCEP3, but SCEP3 alone is not sufficient for SCEP1/SCEP2 localization. Together, while all CR proteins are required for SC assembly, SCEP3 loads independently of other CR components and of SC formation and recruits ZYP1, but SCEP3 is not sufficient for SCEP1/SCEP2 localization.

### SCEP3 and its interaction with ZYP1 are conserved in plants

**Fig. 4:**
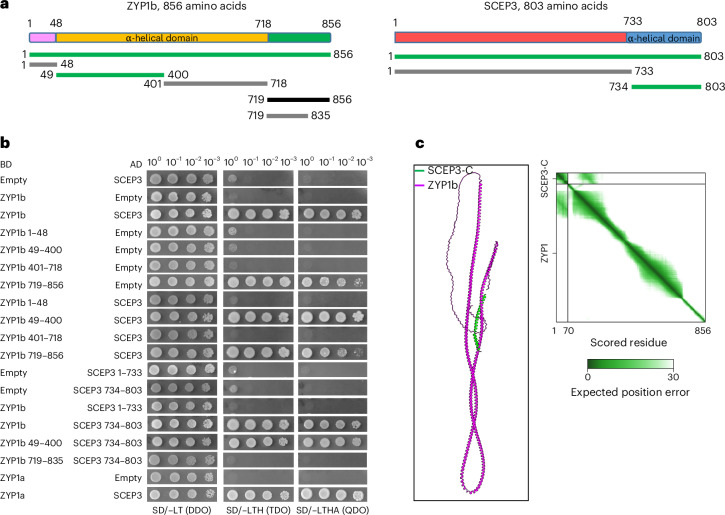
SCEP3 physically interacts with ZYP1. **a**, Schematic depictions of ZYP1b (left) and SCEP3 (right), including fragments tested by Y2H assays. In ZYP1b, the central α-helical domain (orange) is flanked by two flexible regions (pink and green). SCEP3 consists of the C-terminal α-helical domain (blue) and the N-terminal disordered region (red). Green, grey and black lines indicate positive interaction, negative interaction and self-activation, respectively, in the Y2H experiments depicted below. **b**, Y2H interaction studies of ZYP1 and SCEP3. Both ZYP1a and ZYP1b interact with SCEP3 (full-length proteins). To determine the subregion within ZYP1 and SCEP3 responsible for their interaction, ZYP1 (ZYP1b was used) was divided into four fragments and SCEP3 into two. The N-terminal region of ZYP1 (amino acids 49–400) and the C-terminal region of SCEP3 (amino acids 734–803) were found responsible for the interaction between SCEP3 and ZYP1. Selection with minimal SD Base medium supplemented with DO Supplement −Leu/−Trp (SD/−LT (DDO)); DO Supplement −His/−Leu/−Trp (SD/−LT (TDO)); or DO Supplement −Ade/−His/−Leu/−Trp (SD/−LT (QDO)). **c**, AlphaFold3 predicts interaction between the C terminus of SCEP3 and full-length ZYP1b (~100–200 amino acids) in *Arabidopsis*. Predicted aligned error values are shown on the right; the interface predicted template modelling score is 0.48, and the predicted template modelling score is 0.24. Note that AlphaFold’s prediction of the global structure of ZYP1 is not consistent with cytological evidence that the N terminus and C terminus are apart, as previously described^18^.

**Extended Data Fig. 3:**
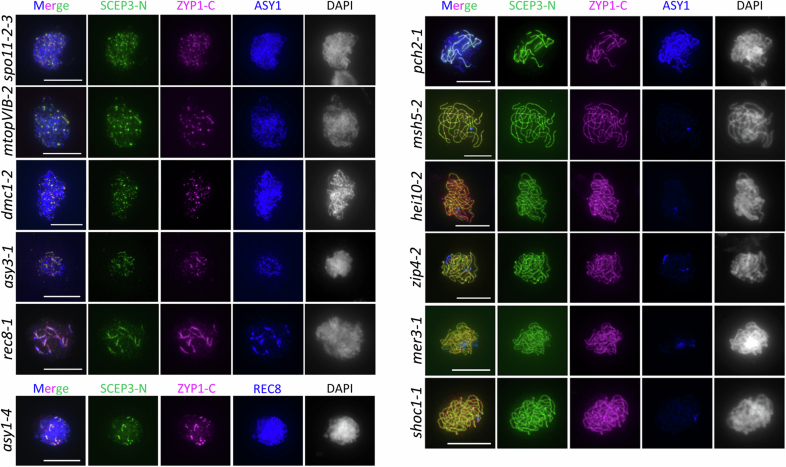
Co-localization of SCEP3 and ZYP1 in various meiotic mutants. Immunolocalization of SCEP3-N, ZYP1-C and ASY1 or REC8 in *spo11-2-3*, *mtopVIB-2*, *dmc1-2*, *asy3-1*, *rec8-1*, *pch2-1*, *asy1-4*, *msh5-2*, *hei10-2*, *zip4-2*, *mer3-1*, and *shoc1-1* pachytene(-like) nuclei. DNA stained with DAPI in gray. All experiments were repeated at least two times with similar results. Scale bar, 10 μm.

Given that SCEP3 and ZYP1 colocalize even in CE mutants, we investigated their relationship in mutants that form an SC despite impaired ZMM-dependent CO formation (*msh5-2*, *hei10-2*, *zip4-2*, *mer3-1* and *shoc1-1*) or exhibit varying degrees of SC formation defects (*spo11-2-3*, *mtopVIB-2*, *dmc1-2*, *asy3-1*, *rec8-1*, *pch2-1* and *asy1-4*). In all cases, SCEP3 and ZYP1 colocalization was found (Extended Data Fig. 3), either as short stretches or fully polymerized within partial or intact SCs, or as foci/protein aggregates in the absence of SC formation. We then tested whether this colocalization reflects a direct interaction. In yeast two-hybrid (Y2H) assays, a strong interaction of SCEP3–ZYP1 (both ZYP1a and ZYP1b) was found, mediated by the N terminus of ZYP1 (ZYP1b, amino acids 49–400) and the C terminus of SCEP3 (amino acids 734–803) (Fig. 4a,b). AlphaFold3 also predicts an interaction between the C-terminal α-helical domain of SCEP3 and the N-terminal region of ZYP1 (Fig. 4c).

**Extended Data Fig. 4:**
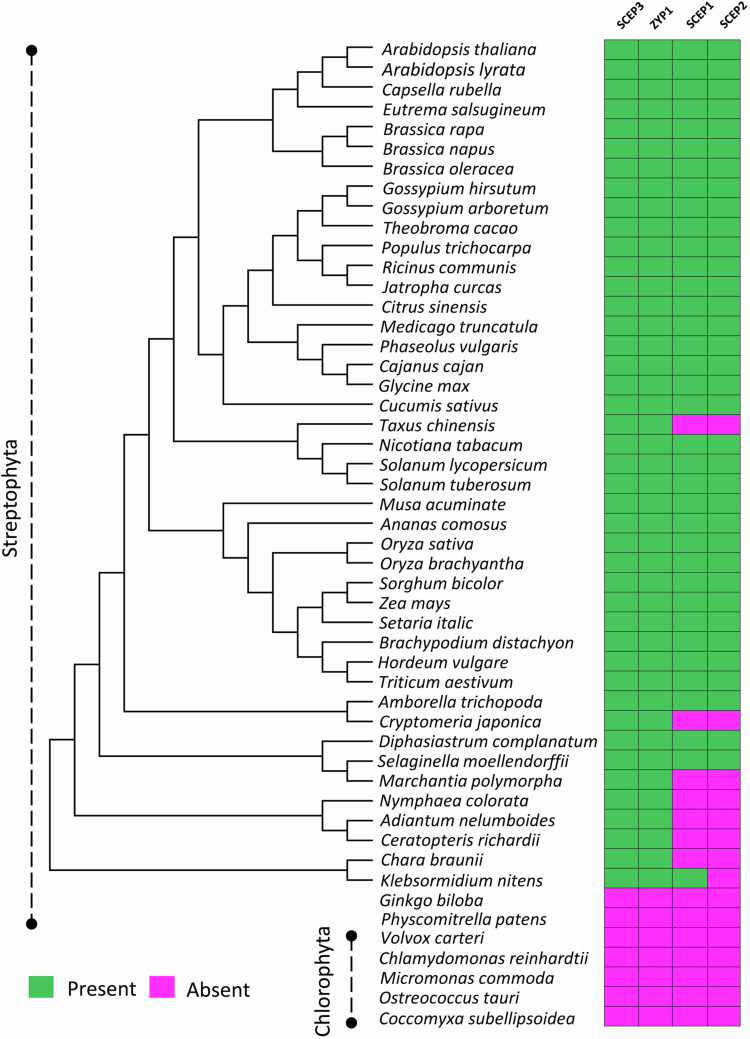
Evolutionary analysis of SCEP3 and other CR components. The evolutionary tree is constructed based on SCEP3 homologous sequences (PSI blast results using Arabidopsis SCEP3 as seed sequence) from Streptophyta species. Accessions used: *A. thaliana* (NP_001328810.1), *A. lyrata* (XP_020875481.1), *Capsella rubella* (XP_023635313.1), *Eutrema salsugineum* (XP_024005618.1), *Brassica rapa* (XP_009136934.2), *B. napus* (XP_013737233.2), *B. oleracea* (XP_013594171.1), *Gossypium hirsutum* (XP_040935483.1), *G. arboretum* (KAK5792206.1), *Theobroma cacao* (XP_017978471.1), *Citrus sinensis* (XP_024950445.1), *Phaseolus vulgaris* (XP_007153704.1), *Cajanus cajan* (XP_029124714.1), *Medicago truncatula* (XP_039689560.1), *Glycine max* (XP_040873294.1), *Ricinus communis* (XP_048229528.1), *Jatropha curcas* (XP_020541047.2), *Populus trichocarpa* (KAI5570781.1), *Cucumis sativus* (XP_011658243.1), *Solanum lycopersicum* (XP_019067843.1), *S. tuberosum* (KAH0717859.1), *Sorghum bicolor* (XP_021312987.1), *Zea mays* (XP_008673176.1), *Setaria italic* (XP_004969993.2), *Oryza sativa* (KAF2952333.1), *O. brachyantha* (XP_040380573.1), *Brachypodium distachyon* (KQK09796.1), *Ananas comosus* (XP_020098929.1), *H. vulgare* (XP_044977082.1), *Nicotiana tabacum* (XP_016437386.1), *Triticum aestivum* (KAF7023752.1), *Musa acuminate* (XP_018686223.1), *Amborella trichopoda* (XP_020527858.1), *Taxus chinensis* (KAH9309445.1), *Selaginella moellendorffii* (XP_024528147.1), *Nymphaea colorata* (XP_049931681.1), *Cryptomeria japonica* (XP_059065081.1), *Adiantum nelumboides* (MCO5557227.1), *Ceratopteris richardii* (KAH7301291.1), *Diphasiastrum complanatum* (KAJ7553908.1), *Marchantia polymorpha* (PTQ45356.1), *Chara braunii* (GBG74560.1), *Klebsormidium nitens* (GAQ79536.1). Some Streptophyta and Chlorophyta species without SCEP3 homolog identified are also listed. The color-coded table includes SCEP3, ZYP1, SCEP1 and SCEP2 and shows the presence (green) or absence (purple) of respective homologs in different species.

**Extended Data Fig. 5:**
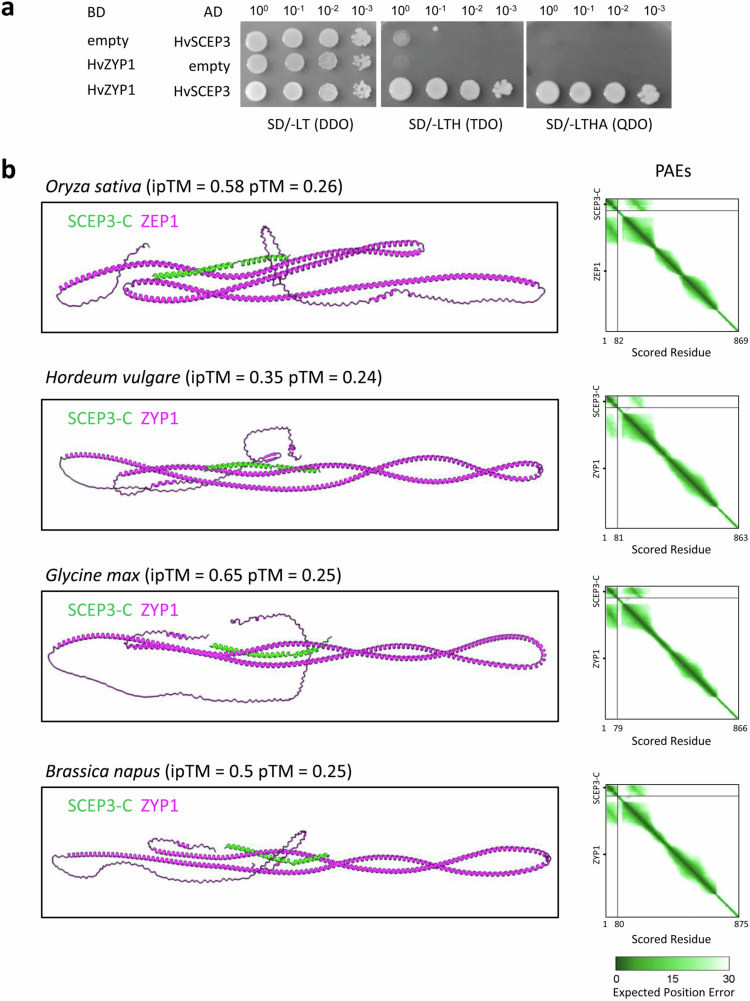
Conservation of the SCEP3 and ZYP1 interaction in plants. **a**, Y2H interaction of *H. vulgare* ZYP1 and SCEP3 (full-length proteins). Note, TDO (SD/-LTH) is a less and QDO (SD/-LTHA) a more stringent medium for selection. **b**, AlphaFold3 interaction prediction using the C-terminus (α-helical domain) of SCEP3 and full-length ZYP1 (ZEP1 is the rice ZYP1 homolog) from *O. sativa*, *H. vulgare*, *G. max* or *B. napus*. PAE values are shown on the right; ipTM and pTM values indicated in parentheses.

Using PSI-BLAST, SCEP3 was found to be conserved across green plants, including many lower plants, but no homologues were identified outside *Streptophyta* (Extended Data Fig. 4). High sequence similarity was found at both the N- and C-terminal regions of SCEP3 (Supplementary Fig. 1). In most plants, homologues of all four CR components are present; however, in lower plants such as *Taxus chinensis* and *Marchantia polymorpha*, homologues of SCEP1/2 (but not SCEP3 and ZYP1) are absent (Extended Data Fig. 4). The close evolutionarily relationship between SCEP3 and ZYP1 may be linked to their direct interaction detected in *A. thaliana*. In Y2H assays, full-length barley SCEP3 and ZYP1 interact (Extended Data Fig. 5a), and AlphaFold3 predicts a conserved SCEP3–ZYP1 interaction across various plants, involving similar regions as in *Arabidopsis* (Extended Data Fig. 5b). Hence, a SCEP3–ZYP1 interface seems highly conserved across plants.

### SCEP3 promotes a subset of COs upon impaired SC formation

**Extended Data Fig. 6:**
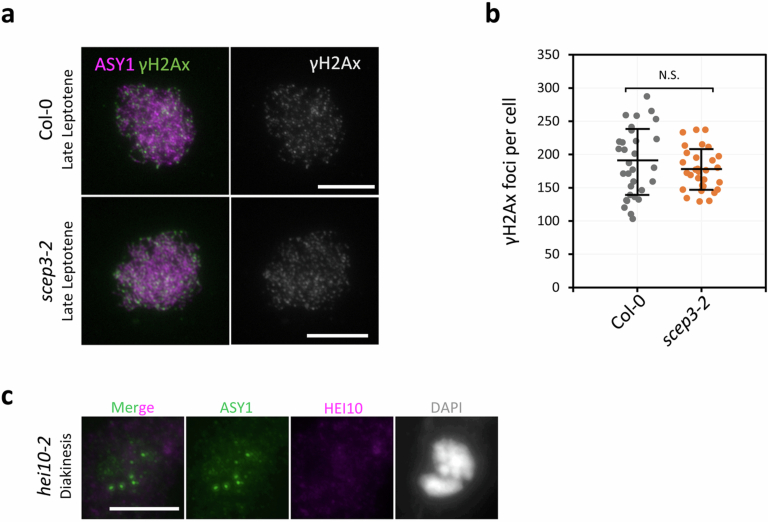
Similar γH2Ax foci numbers in scep3 when compared with the WT and absence of HEI10 immunofluorescence signals in hei10-2. **a**, Immunolocalization of ASY1 and γH2Ax in WT and *scep3-2* meiocytes. γH2Ax signal channel is depicted on the right (gray). Scale bar, 10 μm. **b**, Quantification of γH2Ax foci numbers in Col-0 (187 ± 51 nm, n = 31) and *scep3-2* (177 ± 31 nm, n = 30) meiocytes (late leptotene/early zygotene). Data are presented as mean ± standard deviation (s.d.). No significant difference was found between Col-0 and *scep3-2* (*P* = 0.36, two-sided Student’s *t*-test). N.S., not significant. **c**, Immunolocalization of HEI10 and ASY1 in *hei10-2*. The experiment was repeated two times with similar results. Source data

Using the number of γH2AX foci as a proxy for the number of DSB sites^48,49^, we found no difference between *scep3-2* and the WT (Extended Data Fig. 6a,b). Hence, the shortage of chiasmata in *scep3* is probably not due to reduced DSB numbers.

**Fig. 5:**
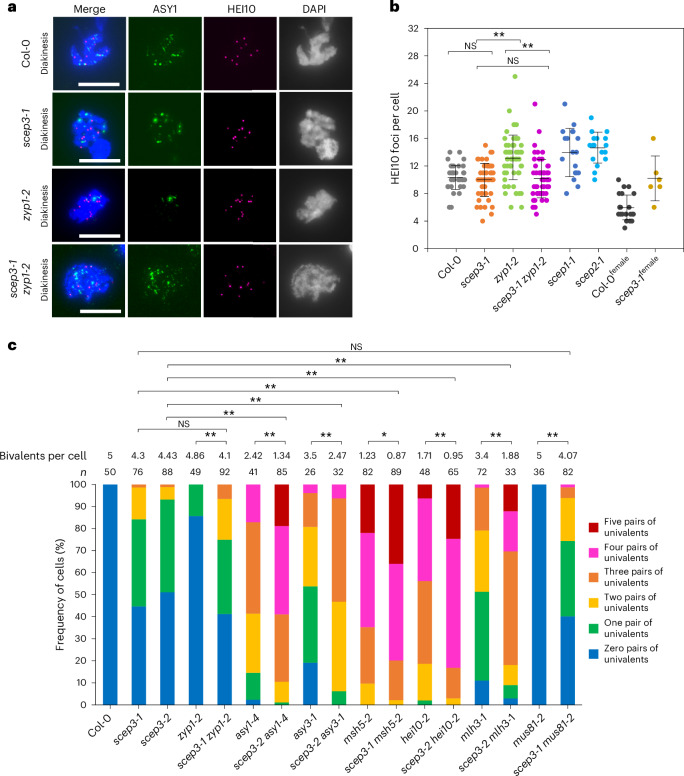
SCEP3 is critical for class I and class II CO formation. **a**,**b**, Immunolocalization of ASY1 and HEI10 (**a**) and quantification of HEI10 foci number per diakinesis cell (**b**) in WT (10.34 ± 1.74, *n* = 41), *scep3-1* (9.94 ± 2.43, *n* = 51), *zyp1-2* (13.18 ± 2.94, *n* = 66), *scep3-1* *zyp1-2* (10.03 ± 2.43, *n* = 59), scep1-1 (13.94 ± 3.62, 18) and scep2-1 (14.59 ± 2.29, *n* = 17) male meiocytes, as well as WT (5.91 ± 1.86, *n* = 23) and *scep3-1* (10.17 ± 3.31, *n* = 6) female meiocytes. Significant differences were found between *scep3-1* and *zyp1-2* (*P* < 1 × 10^−7^) as well as *zyp1-2* and *scep3-1* *zyp1-2* (*P* < 1 × 10^−7^), but not between *scep3-1* and the WT (*P* = 1) or *scep3-1* and *scep3-1* *zyp1-2* (*P* = 1). DNA counterstained with DAPI is shown in merged and single-channel images in blue and grey, respectively. Scale bars, 10 μm. **c**, Frequency of cells with zero to five pairs of univalents including the average bivalent number per cell in a series of single or double mutants. Significant differences were found between *zyp1-2* and *scep3-1* *zyp1-2* (*P* = 2.32 × 10^−7^), *asy1-4* and *scep3-2* *asy1-4* (*P* < 1 × 10^−7^), *scep3-2* and *scep3-2* *asy1-4* (*P* < 1 × 10^−7^), *asy3-1* and *scep3-2* *asy3-1* (*P* = 3.26 × 10^−6^), *scep3-2* and *scep3-2* *asy3-1* (*P* < 1 × 10^−7^), *msh5-2* and *scep3-1* *msh5-2* (*P* = 0.016), *scep3-1* and *scep3-1* *msh5-2* (*P* < 1 × 10^−7^), *hei10-2* and *scep3-2* *hei10-2* (*P* = 4.56 × 10^−7^), *scep3-2* and *scep3-2* *hei10-2* (*P* < 1 × 10^−7^), *scep3-2* and *scep3-2* *mlh3-1* (*P* < 1 × 10^−7^), *mlh3-1* and *scep3-2* *mlh3-1* (*P* < 1 × 10^−7^), and *mus81-2* and *scep3-1* *mus81-2* (*P* < 1 × 10^−7^), but not between *scep3-1* and *scep3-1* *zyp1-2* (*P* = 0.41) or *scep3-1* and *scep3-1* *mus81-2* (*P* = 0.31). Distinct plants from each single or double mutant line (or the WT) were used for immunolocalization and chromosome spread analysis. Significance was assessed using one-way ANOVA with post hoc Bonferroni multiple comparison. The data are presented as mean ± s.d. *P < 0.05; **P < 0.01. Source data

**Extended Data Fig. 7:**
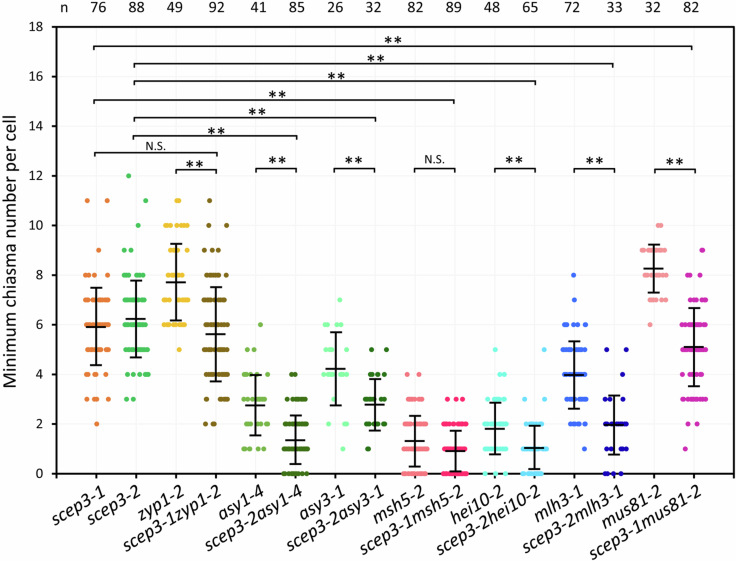
SCEP3 is required for some chiasmata in SC-deficient or ZMM mutants. Minimum chiasma number counts in single (*scep3-1*, *scep3-2*, *zyp1-2*, *asy1-4*, *asy3-1*, *msh5-2*, *hei10-2*, *mlh3-1*, *mus81-2*) and double (*scep3-1zyp1-2*, *scep3-2asy1-4*, *scep3-2asy3-1*, *scep3-1msh5-2*, *scep3-2hei10-2*, *scep3-2mlh3-1*, *scep3-1mus81-2*) mutants. Chromosome spreads were analysed for each mutant using flower buds from distinct plants. The number of cells (n) analysed is indicated at the top. Significance evaluated by one-way ANOVA with post-hoc Bonferroni multiple comparison (*zyp1-2* and *scep3-1zyp1-2*, *P* < 1×10^−7^; *zyp1-2* and *scep3-1zyp1-2*, *P* = 0.71; asy*1-4* and *scep3-2asy1-4*, *P* = 1.96×10^−7^; *scep3-2* and *scep3-2asy1-4*, *P* < 1×10^−7^; asy*3-1* and *scep3-2asy3-1*, *P* = 6.39×10^−4^; *scep3-2* and *scep3-2asy3-1*, *P* < 1×10^−7^; *msh5-2* and *scep3-1msh5-2*, *P* = 0.069; *scep3-1* and *scep3-1msh5-2*, *P* < 1×10^−7^; *hei10-2* and *scep3-2hei10-2*, *P* = 4.72×10^−3^; *scep3-2* and *scep3-2hei10-2*, *P* < 1×10^−7^; *mlh3-1* and *scep3-2mlh3-1*, *P* < 1×10^−7^; *scep3-2* and *scep3-2mlh3-1*, *P* < 1×10^−7^; *mus81-2* and *scep3-1mus81-2*, *P* < 1×10^−7^; *scep3-1* and *scep3-1mus81-2*, *P* = 1.51×10^−3^). Data (refer to Supplemental Table 1) are presented as mean ± standard deviation (s.d.). **P < 0.01. N.S., not significant. Source data

Chiasma counts often underestimate CO numbers, as closely spaced COs cannot be microscopically resolved^16,50^. HEI10 foci undergo a dynamic reduction in number, accompanied by an increase in intensity and size, throughout prophase I. In late pachytene and diakinesis, bright HEI10 foci colocalize with CO-designated sites marked by MLH1 (ref. ^51^). We therefore quantified HEI10 foci as a proxy for class I COs (foci signal is absent in *hei10-2*; Extended Data Fig. 6c). Compared with other CR mutants, which had increased HEI10 foci numbers (13.18 ± 3.28 in *zyp1*, 13.94 ± 3.54 in *scep1* and 14.59 ± 2.29 in *scep2*; Fig. 5a,b), *scep3-1* displayed HEI10 foci counts (9.94 ± 2.43; *P* = 1, one-way analysis of variance (ANOVA) with post hoc Bonferroni multiple comparison) similar to those of the WT (10.34 ± 1.74) (Fig. 5a,b). Thus, in *scep3*, the number of class I COs appears unchanged. However, CO assurance is probably lost due to their random distribution across the genome; other CR mutants also exhibit CO assurance loss, even despite increased HEI10 foci numbers^16–18^. To address whether SCEP3 is required for the surplus HEI10-dependent COs found in *zyp1*, we generated *scep3-1* *zyp1-2* plants. In *scep3-1* *zyp1-2*, the minimum chiasmata number was significantly reduced to 5.62 ± 1.91 compared with 7.69 ± 1.54 in *zyp1-2* (*P* < 0.01, one-way ANOVA with post hoc Bonferroni multiple comparison) but was not significantly different from 5.93 ± 1.56 in *scep3-1* (*P* = 0.71) (Extended Data Fig. 7 and Supplementary Table 1). Univalent frequency in *scep3-1* *zyp1-2* (58.7%) was similar to that in *scep3-1* (55.3%) but higher than that in *zyp1-2* (14.3%) (Fig. 5c). Additionally, HEI10 foci numbers in *scep3-1* *zyp1-2* (10.03 ± 2.80) were similar to those in *scep3-1* (*P* = 1, one-way ANOVA with post hoc Bonferroni multiple comparison) but significantly lower than in *zyp1-2* (13.18 ± 3.28; *P* < 0.01, one-way ANOVA with post hoc Bonferroni multiple comparison) (Fig. 5a,b). Hence, *SCEP3* acts genetically upstream of *ZYP1* in CO formation and is essential for the surplus HEI10-dependent COs observed in *zyp1*. In *asy1* and *asy3*, with impaired SC formation and genetically upstream of *ZYP1* (ref. ^17^), the depletion of *SCEP3* further reduces bivalent/chiasma numbers (Fig. 5c, Extended Data Fig. 7 and Supplementary Table 1). *SCEP3* is thus critical for a subset of COs that arise upon impaired SC formation.

### SCEP3 associates with HEI10 even independent of SC formation

**Fig. 6:**
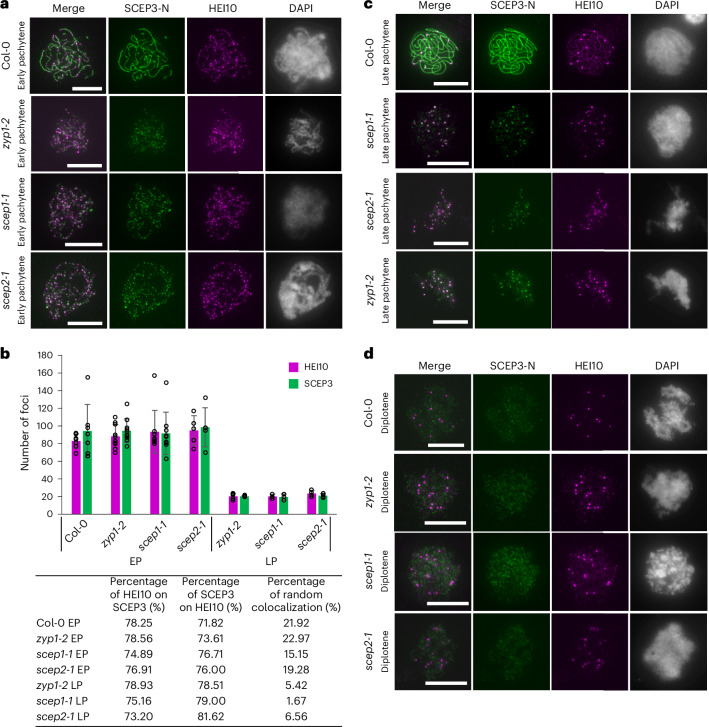
SCEP3 associates with HEI10 independently of SC assembly. **a**, Immunolocalization of SCEP3-N and HEI10 in early pachytene nuclei of the WT, *zyp1-2*, *scep1-1* and *scep2-1*. **b**, Quantification of SCEP3 and HEI10 foci numbers and their percentage of overlap in WT (HEI10, 83 ± 8.1; SCEP3, 94 ± 29.9; *n* = 7), *zyp1-2* (HEI10, 88 ± 13.3; SCEP3, 95 ± 13.4; *n* = 10), *scep1-1* (HEI10, 93 ± 24.2; SCEP3, 92 ± 23.9; *n* = 9) and *scep2-1* (HEI10, 95 ± 16.7; SCEP3, 99 ± 22.1; *n* = 5) early pachytene (EP), as well as *zyp1-2* (HEI10, 21 ± 3.4; SCEP3, 21 ± 0.9; *n* = 5), *scep1-1* (HEI10, 20 ± 2.5; SCEP3, 20 ± 3.5; *n* = 3) and *scep2-1* (HEI10, 24 ± 3.5; SCEP3, 21 ± 2.2; *n* = 4) late pachytene (LP). **c**,**d**, Same as **a**, but in late pachytene and diplotene nuclei, respectively. Distinct plants from each mutant line (or the WT) were used for immunolocalization analysis. DAPI-stained DNA is shown in grey. Scale bars, 10 µm. The data are presented as mean ± s.d. Source data

Since SCEP3 loads and is required for the surplus HEI10-dependent COs in CR mutants (Figs. 3d and 5a,b), we asked whether SCEP3 colocalizes with HEI10. In the WT, during zygotene/early pachytene, discernible SCEP3 foci including those along SCEP3 stretches are closely associated with HEI10 foci, with 78% of HEI10 foci overlapping with SCEP3 foci and 72% of SCEP3 foci overlapping with HEI10 foci (Fig. 6a,b); however, as synapsis progresses, SCEP3 foci gradually transition into continuous linear signals decorated by bright HEI10 foci (Fig. 6c). In *zyp1-2*, *scep1-1* and *scep2-1*, similar rates of association of HEI10 foci with SCEP3 foci (75–79%) and of SCEP3 foci with HEI10 foci (74–77%) are found in early pachytene(-like) nuclei (with ~100 HEI10 foci) (Fig. 6a,b). In late *zyp1-2*, *scep1-1* and *scep2-1* pachytene(-like) nuclei (with ~20 HEI10 foci), although the total numbers of both HEI10 and SCEP3 foci decrease, over 70% of prominent HEI10 foci remain associated with bright SCEP3 foci (Fig. 6b,c). By diplotene/diakinesis, bright HEI10 foci persist at reduced numbers, while bright SCEP3 foci are scarcely detectable (Figs. 5b and 6d). Together, these data suggest an association of SCEP3 and HEI10 throughout pachytene and an enrichment of SCEP3 at putative HEI10-marked CO sites in CR mutants. This association may be required for the surplus HEI10-dependent COs found in these mutants.

**Extended Data Fig. 8:**
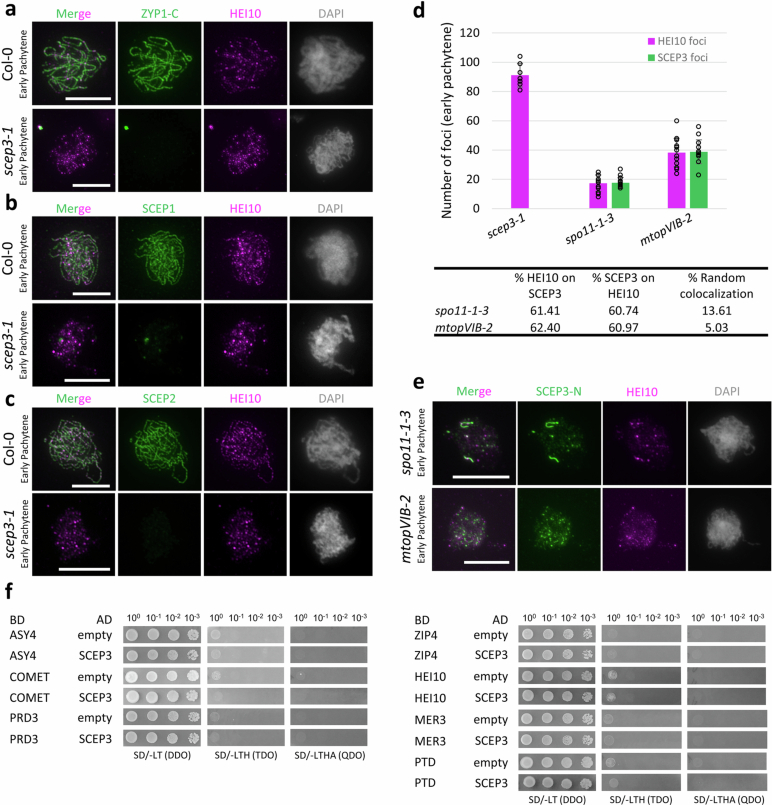
Localization of CR proteins and HEI10 in scep3, spo11-1-3 and mtopVIB-2 as well as no direct interaction of SCEP3 with selected proteins in Y2H. Immunolocalization of **a**, ZYP1-C and HEI10, **b**, SCEP1 and HEI10, and **c**, SCEP2 and HEI10 in early pachytene nuclei of WT and *scep3-1*. **d**, Quantification of SCEP3 and HEI10 foci numbers in *scep3-1* (HEI10, 91 ± 8.1; n = 7), *spo11-1-3* (HEI10, 17 ± 5.6; SCEP3, 18 ± 3.6; n = 15), and *mtopVIB-2* (HEI10, 38 ± 9.7; SCEP3, 39 ± 8.1; n = 14) as well as their percentage of overlap in *spo11-1-3* and *mtopVIB-2*. Data are presented as mean ± standard deviation (s.d.). **e**, Immunolocalization of SCEP3 and HEI10 in early pachytene nuclei of *spo11-1-3* and *mtopVIB-2*. DAPI-stained DNA in gray. Scale bar, 10 µm. **f**, Interactions tested between SCEP3 and axis(-associated) (ASY4, COMET or PRD3) or ZMM proteins (ZIP4, HEI10, MER3 or PTD) in Y2H. TDO (SD/-LTH) is a less stringent and QDO (SD/-LTHA) is a more stringent medium used for selection. The experiments (**a**-**c**, **e**) were repeated at least three times with similar results. Source data

In *scep3-1*, the number of HEI10 foci is similar to that observed in the WT and other CR mutants in early pachytene(-like) nuclei (Fig. 6a,b and Extended Data Fig. 8a–d), suggesting that early HEI10 localization is independent of all CR components. Additionally, ZYP1, SCEP1 and SCEP2 are absent in *scep3-1* (Extended Data Fig. 8a–c). It is therefore tempting to speculate that SCEP3 may associate with a subset of HEI10-marked recombination intermediates, even independent of other CR proteins and SC formation. We noted that, similar to SCEP3 and ZYP1 (Extended Data Fig. 3), HEI10 was found in *spo11-1-3* and *mtopVIB-2* nuclei (Extended Data Fig. 8e). In these backgrounds, SCEP3 and HEI10 are found in association, but at lower numbers and frequencies than in the WT and CR mutants (Fig. 6a,b and Extended Data Fig. 8a–e). They even form aggregates or short stretches (Extended Data Fig. 8e), a pattern not observed in any CR mutant (Fig. 6a). This suggests that SCEP3 and HEI10 are expressed independently of meiotic DSB formation and have an intrinsic tendency to associate, even in the absence of meiotic recombination. Nonetheless, given that SCEP3 is required for the surplus HEI10-dependent COs in *zyp1*, colocalizes with HEI10 in both WT and SC-deficient cells, and is found at putative HEI10-marked CO sites during late pachytene in CR mutants, we propose that SCEP3 may associate with at least a subset of HEI10-marked recombination intermediates.

We next tested whether SCEP3’s axis localization or its spatiotemporal association with HEI10 might reflect a direct interaction with axis(-associated) or ZMM proteins. Interaction between SCEP3 and the axis proteins ASY1, ASY3 and REC8 was not found in Y2H^45^. In addition, we did not detect an interaction between SCEP3 and the other axis(-associated) proteins ASY4, COMET and PRD3, or between SCEP3 and the ZMM proteins ZIP4, HEI10, MER3 and PTD (Extended Data Fig. 8f). However, the absence of a Y2H interaction does not completely rule out the possibility of an interaction in planta.

### SCEP3 is involved in both class I and class II CO formation

We further investigated the role of SCEP3 in CO formation. In mutants impaired in class I CO formation (*msh5-2*, *hei10-2* and *mlh3-1*), SCEP3 depletion further reduces both chiasma and bivalent numbers (Fig. 5c, Extended Data Fig. 7 and Supplementary Table 1; reductions are significant in all cases except *scep3-1* *msh5-2* chiasmata). In addition, the depletion of SCEP3 in *mus81-2* reduces both chiasma (not significantly) and bivalent (significantly) numbers compared with *scep3-1* (Fig. 5c, Extended Data Fig. 7 and Supplementary Table 1). These data suggest that the majority of COs in *scep3* are ZMM-dependent class I COs, while a small fraction rely on MUS81. Importantly, SCEP3 is required for some class II COs, at least in a *zmm* background, as well as for the surplus HEI10-dependent class I COs in the absence of the SC in CR mutants.

### Heterochiasmy and CO interference are abolished in *scep3*

**Extended Data Fig. 9:**
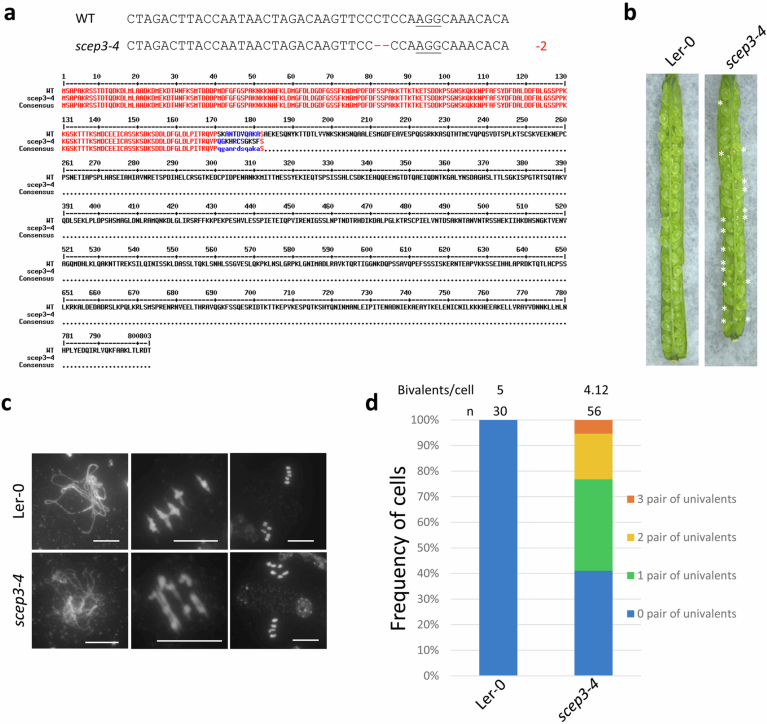
Isolation of scep3-4 in Ler-0. **a**, Details on the CRISPR/Cas9-mediated allele *scep3-4*: 2 bp deletion within exon five resulting in a predicted truncated protein of 182 AA. PAM sequence underlined and deleted bases as red dashes. Below: alignment of SCEP3 wildtype and truncated SCEP3-4 protein. **b**, Seed setting in siliques of Ler-0 and *scep3-4*. Seed abortion indicated by asterisks. **c**, Male meiotic chromosome spreads and **d**, quantification of univalent frequency in Ler-0 and *scep3-4*. Source data

To dissect genome-wide male and female CO events, we isolated *scep3-4* in Ler-0, with similar phenotypes as *scep3* in Col-0 (Fig. 1 and Extended Data Fig. 9). By crossing *scep3-2*^+/−^ with *scep3-4*^+/−^, we generated F_1_ hybrids of the WT (Col-0 × Ler-0) and *scep3* (Col-0 × Ler-0). These hybrids were backcrossed with Col-0 as either the female or male parent, and the resulting four offspring groups (WT female/male and *scep3* female/male) were sequenced.

**Fig. 7:**
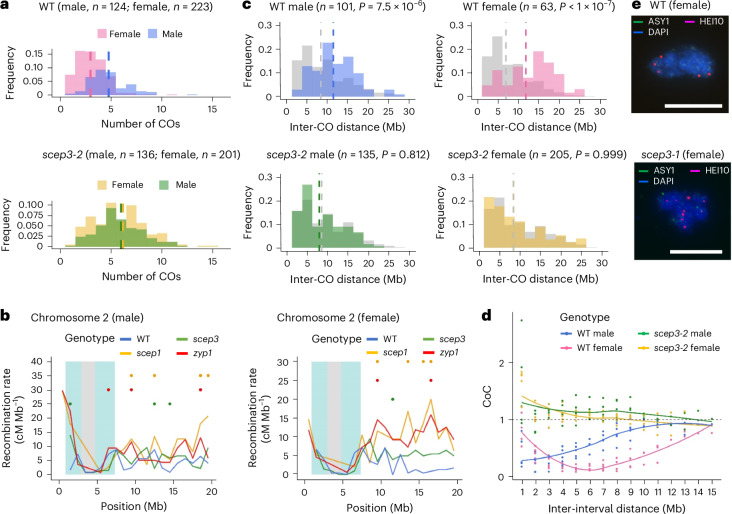
CO interference and heterochiasmy vanish in *scep3.* **a**, Number of COs (the data are presented as mean ± s.e.m.) detected by sequencing of recombinant offspring of WT males (4.80 ± 0.17), WT females (3.00 ± 0.11), *scep3-2* males (6.00 ± 0.24) and *scep3-2* females (6.15 ± 0.23). The sample sizes used for analysis are indicated in parentheses. Significant differences were detected between WT males and females (*P* < 1 × 10^−7^), but not between *scep3-2* males and females (*P* = 0.942) (nested ANOVA followed by Tukey’s honestly significant difference test, a two-sided test with adjustment for multiple comparisons). Compared with the WT, in *scep3-2* CO numbers significantly increased in both males (*P* = 5.09 × 10^−4^) and females (*P* < 1 × 10^−7^). **b**, CO distribution along chromosome 2 in male and female WT, *zyp1* (ref. ^16^), *scep1-1* (ref. ^18^) and *scep3-2*. Centromere and pericentromere regions are indicated in grey and blue, respectively. CO data are presented within 1-Mb windows. Significant differences (based on *χ*^2^ tests) between the WT and *scep3-2* (green dots), *scep3-2* and *zyp1* (red dots) and *scep3-2* and *scep1-1* (orange dots) are indicated. **c**, Distribution of inter-CO distances (only chromosomes with exactly two COs are included for analysis) in male and female WT and *scep3-2*. Calculated random distributions are shown in grey. The statistical significance is indicated in parentheses (nested ANOVA followed by Tukey’s honestly significant difference test, a two-sided test with adjustment for multiple comparisons). **d**, The coefficient of coincidence (CoC) was calculated for inter-interval distances ranging from 1 Mb to 15 Mb for each chromosome, and a LOESS curve was fitted (coloured lines). **e**, Immunolocalization of ASY1 and HEI10 in WT and *scep3-1* female meiocytes. DAPI-stained DNA is shown in blue. Scale bars, 10 μm. Source data

**Extended Data Fig. 10:**
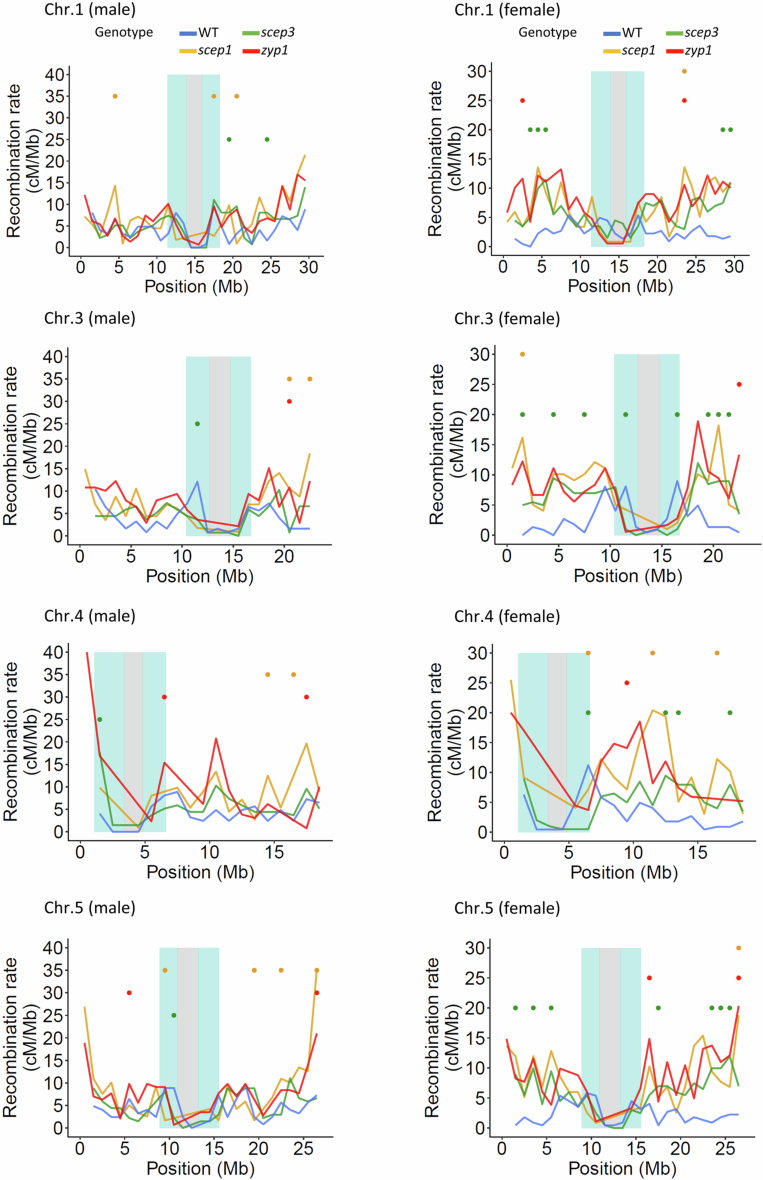
Genome-wide CO distribution in WT and scep3. CO distribution along chromosomes 1, 3, 4 and 5 in male and female of WT, *zyp1*^16^, *scep1-1*^18^ and *scep3-2* plants (refer to Fig. 7b).

In *scep3*, CO numbers significantly increased compared with the WT, by ~25% in males and ~105% in females (Fig. 7a). Heterochiasmy present in the WT vanished in *scep3* (Fig. 7a). CO frequencies increased along chromosome arms, particularly towards chromosome ends, while decreasing in pericentromeric regions (Fig. 7b and Extended Data Fig. 10). In both sexes, CO interference present in the WT was abolished in *scep3* (Fig. 7c,d). Despite comparable CO distributions (Fig. 7b and Extended Data Fig. 10), male and female *scep3* CO levels were ~15% lower than those in *zyp1* or *scep1* (Fig. 7a)^16,18^. In *scep3* males, this is consistent with the absence of increased HEI10 foci and the lower chiasma count than in *zyp1* (Fig. 5b and Extended Data Fig. 7). In contrast, *scep3* females exhibited a striking ~72% increase in HEI10 foci (10.17, *n* = 6) compared with the WT (5.91, *n* = 23) (Figs. 5b and 7e), largely explaining the female CO increase. Overall, CO numbers in *scep3* offspring increased genome-wide, whereas heterochiasmy (primarily due to increased female CO levels) and CO interference vanished, similar to other CR mutants^16,18^.

## Discussion

Two plant CE proteins, SCEP1/2, were previously identified via transcriptomics^18^. We identified SCEP3 using TbID-based proteomics^45^. SCEP3 colocalizes with other CR components at the SC, is essential for SC assembly and interacts with the N terminus of ZYP1. In addition, both the N and C termini of SCEP3 are found at the centre of the SC. Taken together, these findings indicate that SCEP3 is a plant CE protein.

Spatiotemporal and functional overlap among CR proteins suggests potential interactions. SCEP1 and SCEP2 form a complex, yet neither interacts with ZYP1 (ref. ^18^). SCEP3 loads independently of SC formation and other CR proteins. In the absence of the SC, its localization is sufficient for the recruitment of ZYP1, but not for that of SCEP1 or SCEP2. SCEP3 also directly interacts with ZYP1, and this interaction appears to be conserved across plants. Thus, SCEP3 probably acts as a synapsis initiation factor, facilitating ZYP1 recruitment for synapsis initiation. Whether SCEP3 and ZYP1 load as a complex or SCEP3 recruits ZYP1 remains unclear. We prefer the latter possibility, as SCEP3 loads in *zyp1*. Which factor recruits SCEP3 remains unknown, as none of the tested axis or ZMM candidates directly interact with SCEP3 (at least in Y2H) or are critical for its localization. In budding yeast, Zip4 links recombination intermediates with SC assembly by recruiting and interacting with Ecm11 (ref. ^43^), and this mechanism seems conserved in mammals. In worms, SYP-4’s C terminus regulates CO formation, possibly through ZHP-3 (HEI10 orthologue) recruitment^44^. In *Arabidopsis*, the CE proteins SCEP1, SCEP2 (ref. ^18^) and SCEP3 do not directly interact with ZIP4 or HEI10 (at least in Y2H), nor is SC formation impaired in *zip4* or *hei10*. SCEP1 and/or SCEP2 do not form a complex with SCEP3 and/or ZYP1, at least in the absence of the SC; only SCEP3 loads in *zyp1*, and only SCEP3–ZYP1 loads in *scep1* or *scep2* (ref. ^18^). Whether further proteins or modifications and/or the SC context are required for complex formation remains unclear. The *Arabidopsis* SC seems to be composed of at least two subdomains: SCEP3–ZYP1 and SCEP1–SCEP2. However, how they are functionally connected remains to be addressed.

In *scep3*, CO interference, CO assurance and heterochiasmy (primarily due to increased female COs) are lost, suggesting that these are the common phenotypes associated with SC depletion in CR mutants in *Arabidopsis*^16–18^. By contrast, in *scep3*, CO numbers (both male and female) are lower than in the other CR mutants. In *scep3* females, the increase in CO numbers is largely ZMM-dependent (it results from additional HEI10 foci), while no additional HEI10 foci are found in *scep3* males. In both *scep3* sexes, HEI10 foci represent ~83% of the total COs in the offspring, suggesting that ~17% of COs are ZMM-independent class II COs. It is worth noting that the CO rates of *scep3* offspring might be slightly overestimated, as meiotic cells with comparatively high CO numbers may preferentially form viable gametes in *scep3*. Although the majority of COs in *scep3* are class I, SCEP3 is involved in the regulation of both class I and II CO formation. In males, SCEP3 is required for the surplus HEI10-dependent class I COs in *zyp1*, for a proportion of class II COs that arise ZMM-independently, and for some chiasmata in *asy1* and *asy3*, which are impaired in SC formation. Despite no direct interaction in Y2H, SCEP3 associates with HEI10 in both WT and SC-deficient cells. Given its requirement for surplus HEI10-dependent COs in *zyp1*, we propose that the association of SCEP3 and HEI10 foci is probably required for the surplus HEI10-dependent COs found in CR mutants, although HEI10 and an association with SCEP3 are also found in cells deficient for meiotic DSB formation. SCEP3 and HEI10 foci also form exclusively—for example, SCEP3 may also localize at non-ZMM intermediates, as suggested by its requirement for some class II COs. We speculate that SCEP3’s association with certain recombination intermediates before synapsis, and independently of other CR proteins, may stabilize these intermediates and/or recruit additional factors, such as ZYP1, to a subset of them, thereby promoting synapsis initiation and the formation of designated class I and/or II COs.

In *Arabidopsis*, CO interference vanishes in CR mutants^16–18^ including *scep3*, suggesting that the SC per se is required for CO interference implementation. Our data are compatible with the HEI10 coarsening model in which the SC (the scaffold for HEI10 diffusion and condensation) is required for imposing CO interference^40,41,52^. Two non-exclusive scenarios may also contribute to increased CO numbers without signatures of CO interference in CR mutants—that is, longer axis persistence of ASY1 and/or lack of PCH2 (ref. ^53^) or absence of SC polymerization-mediated local downregulation of de novo DSBs^54,55^.

We identified a direct interaction, which seems conserved across plants, between SCEP3’s α-helical C terminus and ZYP1, suggesting that the C terminus of SCEP3 is probably responsible for recruiting ZYP1 to facilitate SC assembly. However, the functional importance of its conserved N terminus remains unclear. The N terminus of SCEP3 is largely disordered, a feature also found in other non-plant CE proteins, such as Ecm11 (yeast), SYP-4 (worms), Corolla (flies) and SIX6OS1 (mammals) (Supplementary Fig. 2). Recently, phenylalanine clusters in the C-terminal disordered region of SYP-4 (which has an α-helical domain at its N terminus) were found to be crucial for CO regulation^44^. SCEP3 also contains phenylalanine clusters at its N terminus, where phenylalanine makes up 12% of the conserved domain, compared with 2.5% in the whole protein, a situation reminiscent of SYP-4’s C terminus. These structural similarities between SCEP3 and SYP-4 strongly suggest that SCEP3 is probably the orthologue of SYP-4 and, by extrapolation, of SIX6OS1 (ref. ^44^). Whether the phenylalanine clusters in SCEP3 share a conserved function with those in SYP-4 is unclear.

We propose a dual role for SCEP3, one as a CR component of the SC, required for its assembly, and the other as a synapsis initiation factor, possibly associated with recombination intermediates. Together, these roles interlink SC and CO formation.

## Methods

### Plant materials and growth conditions

*A. thaliana* plants were grown under short-day conditions (8/16 h light/dark) for four weeks followed by long-day conditions (16/8 h light/dark) until maturity at constant 22 °C. Col-0 was used as the WT except where indicated. The transfer DNA insertion mutants, provided by the Nottingham Arabidopsis Stock Centre^56^, used in this study are *scep3-1* (AT4G18490; SAILseq_210_G05), *scep3-2* (AT4G18490; SALK_098044), *spo11-1-3* (AT3G13170; SALK_146172)^57^, *spo11-2-3* (AT1G63990; GABI_749C12)^57^, *mtopVIB-2* (AT1G60460; GABI_314G09)^58^, *msh5-2* (AT3G20475; SALK_026553)^59^, *mus81-2* (AT4G30870; SALK_107515)^9^, *asy1-4* (AT1G67370; SALK_046272)^60^, *dmc1-2* (AT3G22880; SAIL_170_F08)^61^, *asy3-1* (AT2G46980; SALK_143676)^12^, *rec8-1* (AT5G05490; SALK_137095)^62^, *pch2-1* (AT4G24710; SAIL_1187_C06)^60^, *zip4-2* (AT5G48390; SALK_068052)^63^, *hei10-2* (AT1G53490; SALK_014624)^51^, *mer3-1* (AT3G27730; Salk_091560)^64^, *shoc1-1* (AT5G52290; SALK_057589)^65^ and *mlh3-1* (AT4G35520; SALK_015849)^66^. The mutants *zyp1-2* (ref. ^17^), *scep1-1* and *scep2-1* (ref. ^18^) were described previously. The mutants *scep3-3* and *scep3-4* were isolated using CRISPR–Cas9 in this study. Details on the primers used for genotyping are provided in Supplementary Table 2.

### Isolation of *scep3-3* and *scep3-4* using CRISPR–Cas9

Targeted mutagenesis in *Arabidopsis* via CRISPR–Cas9 was performed according to ref. ^67^. pMOD_A0503, pMOD_B2103, pMOD_C0000 and pTRANS_260d (Addgene nos 91013, 91061, 91081 and 91126) were used for assembling CRISPR constructs with *Cas9* and guide RNAs both driven by the *CmYLCV* promoter. Two guide RNAs (#1: 5′-GAGCCAAAGCCAAAATCCATTGG-3′; #2: 5′-AACTAGACAAGTTCCCTCCAAGG-3′) addressing *SCEP3* were Golden Gate assembled in pMOD_B2103. The final expression cassettes assembled in pTRANS_260d were transferred into ecotypes Col-0 and Ler-0 using *Agrobacterium*-mediated transformation via floral dip^68^. On the basis of Sanger sequencing of target sites, *scep3-3* and *scep3-4* were isolated in transgenic lines in the Col-0 and Ler-0 backgrounds, respectively. For primer details, see Supplementary Table 2.

### Y2H assays

Full-length or truncated coding sequences of *Arabidopsis* *SCEP3*, *ZYP1a*, *ZYP1b*, C terminus of *ZIP4* according to ref. ^18^, *HEI10*, *MER3*, *PTD*, *COMET*, *ASY4* and *PRD3* were PCR-amplified using Col-0 flower bud cDNA as the template and cloned into pGBKT7 and/or pGADT7 vectors (Takara) via Gibson Assembly (NEB). Cloning of barley *SCEP3* and *ZYP1* was done accordingly, but using cDNA prepared from barley anthers (cultivar Golden Promise). For primer details, see Supplementary Table 2.

Y2H assays were performed according to the manufacturer’s instructions (Takara). Bait and prey plasmids (empty vectors as controls) were co-transformed into the yeast strain Y2HGold (Takara, 630489) and grown at 30 °C for three to five days on plates with SD Base medium supplemented with DO Supplement −Leu/−Trp (DDO, 630417). Transformed clones underwent selection assays for five days on plates with Minimal SD Base medium supplemented with DO Supplement −His/−Leu/−Trp (TDO, 630419) or DO Supplement −Ade/−His/−Leu/−Trp (QDO, 630428).

### AlphaFold protein structure modelling

The *Arabidopsis* SCEP3 protein structure (Extended Data Fig. 1a) was modelled using ColabFold v.1.5.3 (AlphaFold2 using MMseqs2)^47^. Modelling of protein–protein interactions (Fig. 4c and Extended Data Fig. 5b) and structures of SC proteins from different species (Supplementary Fig. 2) were performed with AlphaFold Server (AlphaFold3)^69^ and depicted with UCSF ChimeraX^70^.

### Generation of polyclonal antibodies

Peptide synthesis and antibody production were performed by LifeTein LLC. The following peptides were selected the for respective *Arabidopsis* proteins and used for immunization: SCEP3-N (amino acids 69–87; C-GSSFKMDMPDFDFSSPAKK) in rat, SCEP3-C (amino acids 756–775; C-KKKHEEAKELLVRAVVDNNK) in rabbit, HEI10 (C-PKDEIWPARQNS, according to ref. ^71^) in rabbit and guinea pig, ZYP1-C (amino acids 833–851 in ZYP1b; C-SANIGDLFSEGSLNPYADD; peptides identical in ZYP1a/b) in rat and guinea pig, and ASY4 (C-AKLPDELDVDVSSDFKGI) and ASY1 (C-SKAGNTPISNKAQPAASRES, according to ref. ^72^) in rabbit and rat. All antibodies were affinity-purified against the synthetic peptide.

### Cytological procedures

Pollen viability was assessed using Alexander’s stain^73^. Briefly, mature pollen grains were released from anthers in Alexander’s stain solution (MORPHISTO, 13441). After 30 min of staining at room temperature, pollen grains were analysed and counted (viable versus not viable) under a light microscope.

Male and female meiotic chromosome spread preparations and minimum chiasmata number counting were performed as described previously^74^. Briefly, young inflorescences were fixed in Carnoy’s solution (ethanol:acetic acid, 3:1, v/v) for at least 24 h at 4 °C, washed in citric buffer (0.01 M, pH 4.5) and dissected under a stereomicroscope. After dissected flower buds (males) and pistils (females) were digested in an enzyme solution (2% (w/v) cellulase R-10 (Duchefa Biochemie, 9012-54-8) and 1% (w/v) pectolyase Y-23 (Duchefa Biochemie, 9033-35-6), in citrate buffer) at 37 °C for ~70 min, the specimens were washed with 70% ethanol, placed on microscopic slides and macerated in a drop of water using fine forceps. Once the preparations had dried, 7 µl of 100% glacial acetic acid was added. The slides were placed in a humid chamber for 10 min, followed by air-drying and DAPI counterstaining.

Immunolocalization was performed as described previously^74,75^. Briefly, ~20 anthers (per slide) were dissected from young, fresh flower buds (0.35–0.55 mm). The anthers were then digested in 10 µl of an enzyme solution (0.1 g of cytohelicase (Sigma, 42613-29-6), 0.375 g of sucrose and 0.25 g of polyvinylpyrrolidone (Sigma, 9003-39-8) in 25 ml of ddH_2_O) on polysine-coated slides for 5 min at 37 °C, cut with a razor blade and squashed with a brass rod; another 10 µl of enzyme solution was then added. The mixture was then incubated at 37 °C for 7 min. After digestion, 20 µl of 1% lipsol (AZLON, 090844) was added and mixed with the cell suspension to spread the chromosomes. After incubation for 6 min at room temperature, 35 µl of 4% paraformaldehyde (Polysciences, 18814-10) was added to cover the cell area in a fume hood, after which the slides were incubated for at least 2 h in a humid chamber. The slides were then air-dried and treated with PBST (0.1% Triton X-100 in 1× PBS) for 5 min, followed by incubation with primary and secondary antibodies. The following primary antibodies and dilutions were used (conditions and concentrations were identical for the WT and mutants): anti-ASY1 (rabbit^10^; 1:2,000), anti-ASY1 (rabbit or rat, this study; 1:200), anti-ZYP1-C (guinea pig^25^; 1:2,000), anti-ZYP1-C (rat or guinea pig, this study; 1:200), anti-REC8 (rabbit^76^; 1:1,000), anti-ASY4 (rat, this study; 1:200), anti-γH2Ax (mouse, Sigma-Aldrich no. 05-636; 1:200), anti-SCEP3-N (rat, this study; 1:100), anti-SCEP3-C (rabbit, this study; 1:100), anti-HEI10 (guinea pig, this study; 1:200), anti-SCEP1 (rabbit^18^; 1:200) and anti-SCEP2 (rat^18^; 1:200). The following secondary antibodies were used (all diluted 1:500): anti-guinea-pig Cy5 (Abcam, ab102372), anti-guinea-pig Alexa 594 (Invitrogen, A11076), anti-guinea-pig Alexa 488 (Invitrogen, A11073), anti-rabbit Alexa 594 (Abcam, ab150076), anti-rabbit Alexa 488 (Abcam, ab150073), anti-rabbit Cy3 (Jackson ImmunoResearch, 111-165-003), anti-rat Alexa 488 (Jackson ImmunoResearch, 112-545-167) and anti-rat Alexa 594 (Abcam, ab150160).

### Microscopy

Epifluorescence images were acquired using a Nikon Eclipse Ni-E microscope equipped with a Nikon DS-Qi2 camera and NIS-Elements-AR v.4.60 software (Nikon). The acquisition parameters were maintained for each antibody combination, independent of the material background (WT versus mutants), and chosen to be those that allowed capturing the most information while avoiding saturation to ensure accurate visualization and quantification. After image acquisition, deconvolution was performed using NIS-Elements-AR v.4.60 software with the method ‘Fast’ and the default parameters.

SCEP3–HEI10 colocalization analysis was performed using Imaris (Bitplane) v.10.1.0. Automatic spot detection with a size of 0.33 µm and background subtraction was applied for the red (HEI10) and the green (SCEP3) channels independently. Afterwards, a filter was chosen on the red channel to identify spots overlapping with spots in the green channel, allowing a maximum distance of 0.33 µm. The frequency of overlapping foci was afterwards obtained by calculating (number of overlapping foci × 100/total of red) and (number of overlapping foci × 100/total of green). To determine randomness of colocalization, the image was rotated 90°, spots were again detected for the green channel with the same parameters and these newly created spots were compared to the original red spots.

3D-SIM image stacks were acquired using an Elyra 7 microscope system and the software ZEN Black 3.0 v.16.0 (Carl Zeiss GmbH)^77^. Inter-axis distances were measured from centre to centre of each parallel axis in single-slice images (the positions were randomly picked).

Two-colour STED images were acquired with Abberior Expert Line with an Olympus UPlanSApo ×100/1.4 oil-immersion objective. Star-Orange and Star-Red were excited by 561-nm and 640-nm pulsed diode lasers, respectively, and the fluorescence signals were inhibited by a pulsed 775-nm depletion laser (total power, 3 W). For STED microscopy to perform at its full potential, alignment of the excitation and depletion beams was needed as described previously^78^. Briefly, the centres of the excitation and depletion beams were overlapped initially by scanning gold beads of 150 nm (BBI Solutions) in a reflection mode. Afterwards, TetraSpeck beads of four colours (TetraSpeckTM Microspheres; 100 nm; fluorescent blue, green, orange and dark red) were used to correct mismatches between the scattering mode and the fluorescence mode. To ensure correct and precise positioning of the same beads imaged by different laser lines, individual confocal and STED microscope channels were compared and adjusted respectively as the last step. During STED microscopy sample imaging, sequential scanning was applied to keep photobleaching of the samples at a minimum state, with first the Star-Red channel and then the Star-Orange channel. The images were acquired with a pixel size of 20 nm and a pixel dwell time of 10 µs, and a distance of 100 nm was applied between each slice for xyz stacks. The images were deconvolved with Huygens Professional v.24.10 (Scientific Volume Imaging, https://svi.nl/) using the classic maximum likelihood estimation algorithm with lateral drift stabilization and with 27 iterations (the detailed parameter selections were based on previously reported values^16^). Maximum intensity projections and contrast adjustments were applied to the deconvolved images using Fiji (open source)^79^. Additional image processing (adjusting brightness/contrast and merging of individual channels) was done with Fiji/ImageJ v.2.9.0 (NIH), Adobe Photoshop CS5 (Adobe) and ZEN v.3.1 (blue edition).

### Genome-wide mapping of male and female COs in the WT and *scep3*

To generate male and female CO mapping populations, *scep3-2*^+/−^ (Col-0) was crossed with *scep3-4*^+/−^ (Ler-0), and F_1_ hybrids of the WT or biallelic for *scep3* were crossed as a male or female with the WT Col-0. Total DNA samples were prepared from the four resulting backcross populations (WT male, 143 plants; WT female, 237 plants; *scep3-2* *scep3-4* male, 142 plants; *scep3-2* *scep3-4* female, 238 plants) using a ~150-mg leaf sample per individual plant and Econospin columns (96 well, Epoch Life Sciences) according to the manufacturer’s instructions adapted to plant samples. Whole-genome shotgun sequencing library preparation (Illumina DNA PCR-Free Library Prep, Tagmentation with standard DNA input amount) involved protocols from the manufacturer (Illumina). The library was quantified using quantitative PCR (KAPA Library Quant Kit; Roche Molecular Systems) and sequenced according to the manufacturer’s instructions using the NovaSeq 6000 device (Illumina; run type, SP flowcell with XP workflow and paired-end sequencing: 151 cycles (read 1) 10 cycles (index read 1), 10 cycles (index read 2) and 151 cycles (read 2)) at IPK Gatersleben.

Raw sequence reads were aligned to the *A. thaliana* Col TAIR10 reference genome^80^ using BWA-MEM^81^, converted to Binary/Alignment Map format, and sorted using SAMtools^82^. Variant calling was done using BCFtools^83^, filtering for a minimum mapping quality and minimum base quality of 30. The resulting variants matrix in Variant Call format was filtered using VCFtools^84^ for biallelic single nucleotide variants (SNVs), a minor allele frequency ranging between 0.2 and 0.3, a minimum read depth per site of 4, a maximum read depth per site of 100, a minimum mean read depth of 1 across all samples and a maximum mean read depth of 1.5. The resulting variant matrix contained 350,575 high-quality SNVs. Individuals with more than 75% missing data were removed (9 out of 760 samples). Genotype calls homozygous for the reference allele (Ler) were removed from further analysis (average of 8% per sample). Samples with SNV numbers below the 5% percentile or above the 95% percentile were removed from further analysis to avoid potential biases caused by extreme marker number deviations. To measure recombination events, SNV information was first aggregated in sliding windows of 20 consecutive SNVs with a step size of 1 by determining the modal SNV. Second, smoothed SNVs were further aggregated in non-overlapping windows of 1 Mb. For CO interference analysis, only chromosomes with exactly two COs were used. Observed inter-CO distances were compared against random inter-CO distances obtained via 500 permutations of the respective dataset (that is, male/female WT or male/female *scep3*). CO positions of *zyp1* and *scep1* were retrieved from refs. ^16,18^. The CoC was calculated in inter-interval distances from 1 Mb to 15 Mb for each chromosome (CoC = observed frequency of double COs / expected frequency of double COs). Statistical analysis of CO count and inter-CO distance was done in R v.4.4.1 via nested ANOVA (aov(CO count or inter-CO distance ~ sex/genotype)) followed by Tukey’s honestly significant difference test. Recombination landscapes were analysed via *χ*^2^ tests.

### Material availability

Generated materials are available from the corresponding author upon reasonable request.

### Reporting summary

Further information on research design is available in the Nature Portfolio Reporting Summary linked to this article.

## Supplementary information

Supplementary Information Supplementary Figs. 1 and 2.

Reporting Summary

Supplementary Data 1 Supplementary Tables 1 and 2.

## Source data

Source Data Figs. 1, 2 and 5–7 and Extended Data Figs. 1 and 6–9 Statistical source data.

## Data Availability

The data supporting the findings of this research are presented in the main text, figures and supplementary information. The whole-genome resequencing raw data underlying Fig. 7 and Extended Data Fig. 10 have been deposited to the European Nucleotide Archive under accession number PRJEB81799 (http://www.ebi.ac.uk/ena/data/view/PRJEB81799). The gene/protein sequences and accession codes used in this study are available in the databases TAIR (https://www.arabidopsis.org/) and Ensembl Plants (http://plants.ensembl.org/index.html). The predicted protein structures are available in the AlphaFold Protein Structure Database (https://alphafold.ebi.ac.uk/). Source data are provided with this paper.
