# Supplementary material for: The synaptonemal complex central element SCEP3 interlinks synapsis initiation and crossover formation in Arabidopsis thaliana
Source: Nat Plants. 2025 Jun 27;11(7):1353–66. doi: 10.1038/s41477-025-02030-9 (PMC12283363; doi:10.1038/s41477-025-02030-9)
Supplement: Supplementary file 1 — Supplementary Figs. 1 and 2. [file 41477_2025_2030_MOESM1_ESM.pdf]

nature plants

Supplementary information https://doi.org/10.1038/s41477-025-02030-9

The synaptonemal complex central element SCEP3 interlinks synapsis initiation and crossover formation in *Arabidopsis thaliana*

In the format provided by the authors and unedited

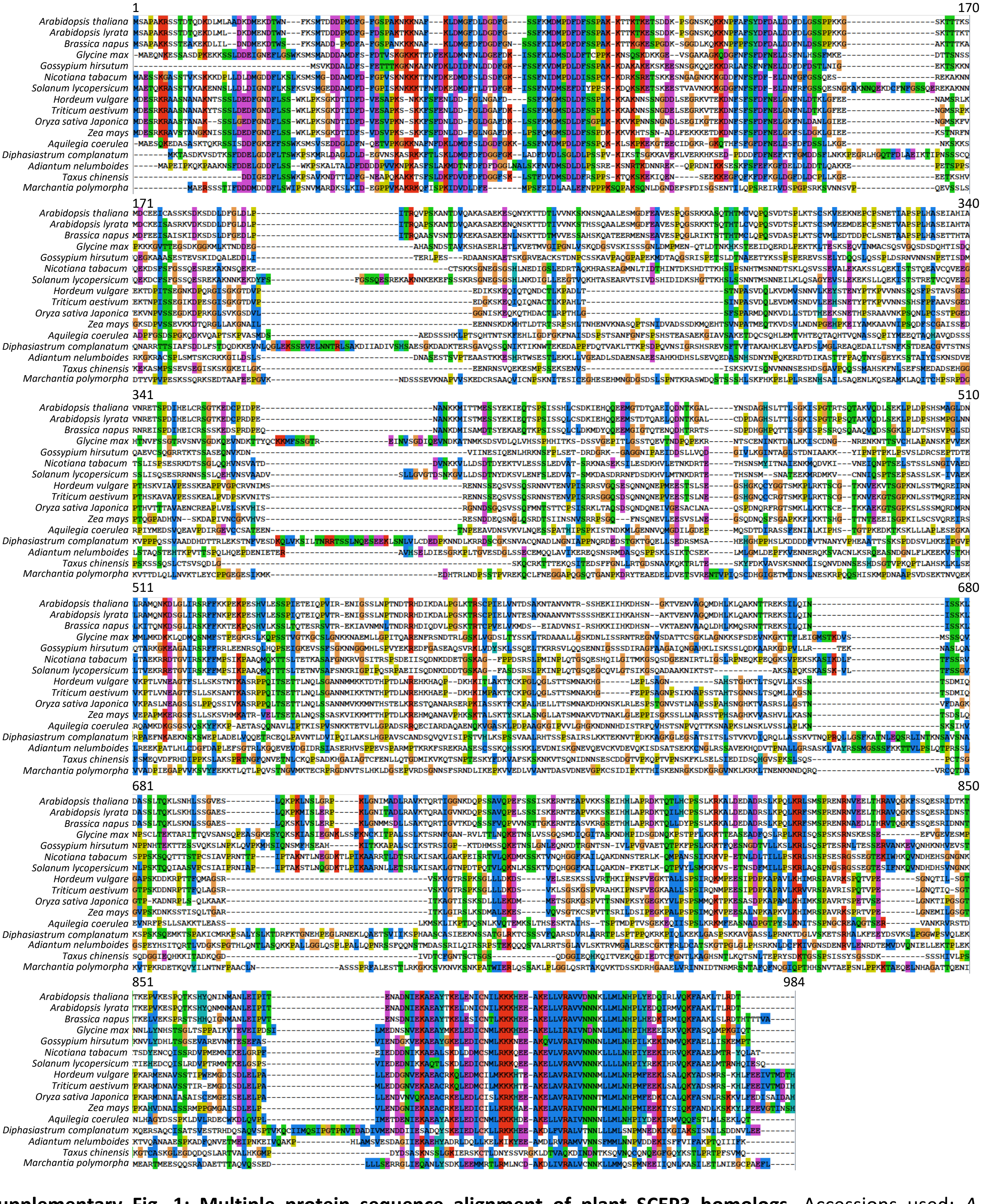

Supplementary Fig. 1: Multiple protein sequence alignment of plant SCEP3 homologs. Accessions used: *A. thaliana*, NP_193584.3; *A. lyrata*, XP_020875478.1; *B. napus*, XP_013737233.1; *G. max*, XP_006583844.1; *G. hirsutum*, KAG4178256.1; *N. tabacum*, XP_016437384.1; *S. lycopersicum*, XP_019067843.1; *H. vulgare*, XP_044977082.1; *T. aestivum*, KAF7030755.1; *O. sativa Japonica*, XP_015621851.1; *Z. mays*, NP_001348911.1; *Aquilegia coerulea*, IA41840.1; *D. complanatum*, KAJ7553908.1; *A. nelumboides*, MCO5557227.1; *T. chinensis*, KAH9309445.1; *M. polymorpha*, PTQ45357.1.

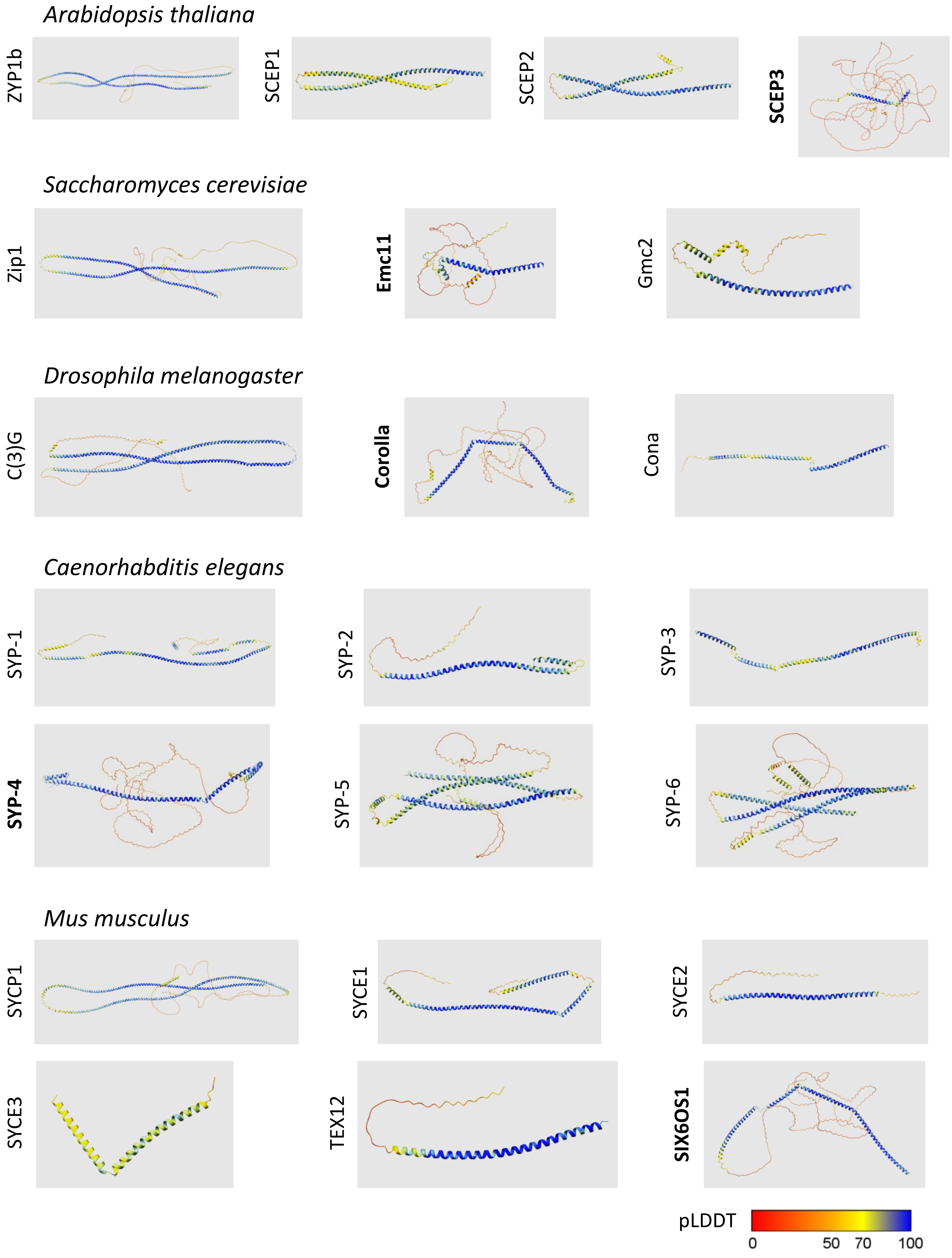

Supplementary Fig. 2: SC proteins from non-plant species: possible structural SCEP3 orthologues. Known SC proteins as potential non-plant SCEP3 structural orthologues isolated from the AlphaFold protein structure database.

85. Jumper J*, et al.* Highly accurate protein structure prediction with AlphaFold. *Nature* **596**, 583-589 (2021).

86. Varadi M*, et al.* AlphaFold Protein Structure Database in 2024: providing structure coverage for over 214 million protein sequences. *Nucleic Acids Res.* **52**, D368-D375 (2024).

85. Jumper J*, et al.* Highly accurate protein structure prediction with AlphaFold. *Nature* **596**, 583-589 (2021).

86. Varadi M*, et al.* AlphaFold Protein Structure Database in 2024: providing structure coverage for over 214 million protein sequences. *Nucleic Acids Res.* **52**, D368-D375 (2024).
