# Supplementary material for: The synaptonemal complex central element SCEP3 interlinks synapsis initiation and crossover formation in Arabidopsis thaliana
Source: Nat Plants. 2025 Jun 27;11(7):1353–66. doi: 10.1038/s41477-025-02030-9 (PMC12283363; doi:10.1038/s41477-025-02030-9)
Supplement: Supplementary file 2 — Reporting Summary [file 41477_2025_2030_MOESM2_ESM.pdf]

nature portfolio

Corresponding author(s): Stefan Heckmann

Last updated by author(s): May 20, 2025

Reporting Summary

Nature Portfolio wishes to improve the reproducibility of the work that we publish. This form provides structure for consistency and transparency in reporting. For further information on Nature Portfolio policies, see our Editorial Policies and the Editorial Policy Checklist.

Statistics

For all statistical analyses, confirm that the following items are present in the figure legend, table legend, main text, or Methods section.

n/a	Confirmed	
☐	☒	The exact sample size (*n*) for each experimental group/condition, given as a discrete number and unit of measurement
☐	☒	A statement on whether measurements were taken from distinct samples or whether the same sample was measured repeatedly
☐	☒	The statistical test(s) used AND whether they are one- or two-sided<br>*Only common tests should be described solely by name; describe more complex techniques in the Methods section.*
☒	☐	A description of all covariates tested
☒	☐	A description of any assumptions or corrections, such as tests of normality and adjustment for multiple comparisons
☐	☒	A full description of the statistical parameters including central tendency (e.g. means) or other basic estimates (e.g. regression coefficient) AND variation (e.g. standard deviation) or associated estimates of uncertainty (e.g. confidence intervals)
☐	☒	For null hypothesis testing, the test statistic (e.g. *F*, *t*, *r*) with confidence intervals, effect sizes, degrees of freedom and *P* value noted<br>*Give P values as exact values whenever suitable.*
☒	☐	For Bayesian analysis, information on the choice of priors and Markov chain Monte Carlo settings
☒	☐	For hierarchical and complex designs, identification of the appropriate level for tests and full reporting of outcomes
☒	☐	Estimates of effect sizes (e.g. Cohen's *d*, Pearson's *r*), indicating how they were calculated

Our web collection on statistics for biologists contains articles on many of the points above.

Software and code

Policy information about availability of computer code

Data collection	Fluorescence images were acquired using Nikon Eclipse Ni-E equipped with a Nikon DS-Qi2 camera and NIS-Elements-AR version 4.60 software (Nikon, Tokyo, Japan). 3D-SIM images were acquired using an Elyra 7 microscope system and the software ZENBlack 3.0 Version 16.0 (Carl Zeiss GmbH). STED images were acquired using Imspector 16.3.16132 software. Genome sequencing data were acquired using NovaSeq 6000 device (Illumina, Inc., San Diego, CA, USA).
Data analysis	Cloning: SnapGene 4.2.4 (GSL Biotech LLC, USA)<br>Statistical analysis: Microsoft Excel 2016 (Microsoft Corporation, USA)<br>Image processing: Adobe Photoshop CS5 (Adobe Inc., USA)); Fiji (open source); ZEN 3.1 (blue edition)<br>Co-localization analysis: Imaris version 10.1.0 (Bitplane, Switzerland)<br>De-convolution (for STED images): Huygens Professional version 24.10 (Scientific Volume Imaging, https://svi.nl/)<br>Protein structure modeling: ColabFold v1.5.3 (AlphaFold2 using MMseqs2) or AlphaFold Server (AlphaFold3)<br>Genome wide crossover mapping: BWA-MEM (arXiv:1303.3997); SAMtools (Gigascience 10:giab008); BCFtools (Bioinformatics 25:2078–2079); VCFtools (Bioinformatics 27:2156–2158)

For manuscripts utilizing custom algorithms or software that are central to the research but not yet described in published literature, software must be made available to editors and reviewers. We strongly encourage code deposition in a community repository (e.g. GitHub). See the Nature Portfolio guidelines for submitting code & software for further information.

Data

Policy information about availability of data

All manuscripts must include a data availability statement. This statement should provide the following information, where applicable:

- Accession codes, unique identifiers, or web links for publicly available datasets
- A description of any restrictions on data availability
- For clinical datasets or third party data, please ensure that the statement adheres to our policy

All data supporting the findings of this research are presentend in the main text, figures and supplementary information. Whole-genome re-sequencing raw data are deposited to the European Nucleotide Archive (ENA) under accession number PRJEB81799 (http://www.ebi.ac.uk/ena/data/view/PRJEB81799). Gene/protein sequences and accession codes used in this study are found in databases TAIR (https://www.arabidopsis.org/) and Ensembl Plants (http://plants.ensembl.org/index.html). Predicted proteins structures are found in AlphaFold Protein Structure Database (https://alphafold.ebi.ac.uk/). Source data are provided with this paper.

Research involving human participants, their data, or biological material

Policy information about studies with human participants or human data. See also policy information about sex, gender (identity/presentation), and sexual orientation and race, ethnicity and racism.

Reporting on sex and gender	N/A
Reporting on race, ethnicity, or other socially relevant groupings	N/A
Population characteristics	N/A
Recruitment	N/A
Ethics oversight	N/A

Note that full information on the approval of the study protocol must also be provided in the manuscript.

Field-specific reporting

Please select the one below that is the best fit for your research. If you are not sure, read the appropriate sections before making your selection.

☒ Life sciences ☐ Behavioural & social sciences ☐ Ecological, evolutionary & environmental sciences

For a reference copy of the document with all sections, see nature.com/documents/nr-reporting-summary-flat.pdf

Life sciences study design

All studies must disclose on these points even when the disclosure is negative.

Sample size	No sample-size calculations were performed. Sample sizes were chosen based on our previous experiences on similar experiments in the lab, based on published work (e.g. Osman et al. 2018, Cuacos et al. 2021, Feng et al. 2023) and based on the availability of samples. Sample sizes are indicated throughout the manuscript and were sufficient to result in scientific significance as well as reproducibility of acquired results.
Data exclusions	No data were excluded.
Replication	All data were replicable and the number of replicates performed are indicated in the main text, figure captions or the Methods.
Randomization	For all studies, plants of different genotypes were randomized and grown under controlled conditions to avoid environmental effects.
Blinding	All experiments were performed without prior knowledge of the outcome and hence no blinding was performed in the experiments.

Reporting for specific materials, systems and methods

We require information from authors about some types of materials, experimental systems and methods used in many studies. Here, indicate whether each material, system or method listed is relevant to your study. If you are not sure if a list item applies to your research, read the appropriate section before selecting a response.

Materials & experimental systems

n/a	Involved in the study	
☐	☒	Antibodies
☒	☐	Eukaryotic cell lines
☒	☐	Palaeontology and archaeology
☒	☐	Animals and other organisms
☒	☐	Clinical data
☒	☐	Dual use research of concern
☐	☒	Plants

Methods

n/a	Involved in the study	
☒	☐	ChIP-seq
☒	☐	Flow cytometry
☒	☐	MRI-based neuroimaging

Antibodies

Antibodies used	Primary antibodies:<br>Anti-ASY1 (rabbit, 1:2000, J. Cell Sci. 115, 3645-3655), anti-ZYP1 (guinea pig, 1:2000, Genes Dev. 19, 2488-2500) are gifts kindly provided by Prof. Chris Franklin (University of Birmingham). Anti-REC8 (rabbit, 1:1000, Curr. Biol. 23, 2090-2099), anti-SCEP1 and anti-SCEP2 (rat, 1:200, Nat. Plants 9, 2016–2030) are kindly provided by Dr. Mathilde Grelon. Anti-ASY1 (rabbit or rat, 1:200), anti-ZYP1-C (rat or guinea pig, 1:200), anti-SCEP3-N (rat, this study; 1:100), anti-SCEP3-C (rabbit, this study; 1:100), anti-ASY4 (rat, this study; 1:200) and anti-HEI10 (guinea pig, 1:200) are generated in this study. Other antibodies used are commercially available: anti-γH2Ax (mouse, 1:200; Sigma-Aldrich #05-636).<br>Secondary antibodies (diluted in 1:500):<br>Anti-guinea pig Cy5 (Abcam, ab102372), anti-guinea pig Alexa 594 (Invitrogen, A11076), anti-guinea pig Alexa 488 (Invitrogen, A11073), anti-rabbit Alexa 594 (Abcam, ab150076), anti-rabbit Alexa 488 (Abcam, ab150073), anti-rabbit Cy3 (Jackson ImmunoResearch, 111-165-003), anti-rat Alexa 488 (Jackson ImmunoResearch, 112-545-167) and anti-rat alexa 594 (Abcam, ab150160). For STED microscopy, secondary antibodies labeled with Abberior STAR ORANGE or STAR RED were used.
Validation	Validation of some of the primary antibodies used in this study are found in the following websites or publications:<br>anti-ASY1 (rabbit) and anti-ZYP1-C (guinea pig) (https://doi.org/10.1371/journal.pgen.1002507)<br>anti-REC8 (https://www.cell.com/current-biology/fulltext/S0960-9822(13)01042-7)<br>anti-SCEP1 and anti-SCEP2 (https://www.nature.com/articles/s41477-023-01558-y)<br>anti-γH2Ax (https://www.sigmaaldrich.com/DE/de/product/mm/05636)<br>Other antibodies generated in this study:<br>anti-SCEP3-N and anti-SCEP3-C were validated by immunolocalization and shown in Extended Data Fig. 2<br>anti-HEI10 was validated by immunolocalization and shown in Fig. 5 and Extended Data Fig. 6<br>anti-ASY1 (rabbit or rat), anti-ZYP1-C (rat or guinea pig) and anti-ASY4 were validated by immunolocalization and shown in Fig. 2, 3

Dual use research of concern

Policy information about dual use research of concern

Hazards

Could the accidental, deliberate or reckless misuse of agents or technologies generated in the work, or the application of information presented in the manuscript, pose a threat to:

No	Yes	
☒	☐	Public health
☒	☐	National security
☒	☐	Crops and/or livestock
☒	☐	Ecosystems
☒	☐	Any other significant area

Experiments of concern

Does the work involve any of these experiments of concern:

No	Yes	
☒	☐	Demonstrate how to render a vaccine ineffective
☒	☐	Confer resistance to therapeutically useful antibiotics or antiviral agents
☒	☐	Enhance the virulence of a pathogen or render a nonpathogen virulent
☒	☐	Increase transmissibility of a pathogen
☒	☐	Alter the host range of a pathogen
☒	☐	Enable evasion of diagnostic/detection modalities
☒	☐	Enable the weaponization of a biological agent or toxin
☒	☐	Any other potentially harmful combination of experiments and agents

Plants

Seed stocks

scep3-1(N809841), scep3-2(N598044), spo11-1-3 (N646172), msh5-2 (N526553), mus81-2(N607515), asy1-4(N546272), dmc1-2(N871769), asy3-1(N643676), rec8-1(N637095), pch2-1(N861552), zip4-2(N568052), hei10-2(N514624), mer3-1(N591560), shoc1-1(N557589) and mlh3-1(N515849) were provided by the NASC (https://arabidopsis.info). spo11-2-3 (Plant Cell 19, 3090-3099), mtopVIB-2 (Science 351, 939-943), zyp1-2 (PNAS 118 (14), e2021671118), scep1-1 and scep2-1 (Nat. Plants 9, 2016–2030) were described.

Novel plant genotypes

The novel alleles scep3-3 (Col-0) and scep3-4 (Ler-0) were isolated using CRISPR/Cas9 targeting exon 5 (target sequence: AACTAGACAAGTTCCCTCCAAGG) of SCEP3. The CRISPR/Cas9 construct was transformed into Arabidopsis via Agrobacterium-mediated transformation by floral dip. The following alleles are generated by crossing: scep3-1scep3-2, scep3-2scep3-4, scep3-1zyp1-2, scep3-2asy1-4, scep3-2asy3-1, scep3-1msh5-2, scep3-2hei10-2, scep3-2mlh3-1 and scep3-1mus81-2.

Authentication

All T-DNA insertion lines used in this study were confirmed by PCR with specific primers (including negative controls). Allelism between scep3-1 and scep3-2 was confirmed. The two CRISPR alleles scep3-3 and scep3-4 were confirmed by Sanger sequencing. Comparable phenotype of all alleles scep3 (-1 to -4) were confirmed.
